# Reconstruction of hadronic decay products of tau leptons with the ATLAS experiment

**DOI:** 10.1140/epjc/s10052-016-4110-0

**Published:** 2016-05-25

**Authors:** Atlas Collaboration, G. Aad, B. Abbott, J. Abdallah, O. Abdinov, R. Aben, M. Abolins, O. S. AbouZeid, H. Abramowicz, H. Abreu, R. Abreu, Y. Abulaiti, B. S. Acharya, L. Adamczyk, D. L. Adams, J. Adelman, S. Adomeit, T. Adye, A. A. Affolder, T. Agatonovic-Jovin, J. Agricola, J. A. Aguilar-Saavedra, S. P. Ahlen, F. Ahmadov, G. Aielli, H. Akerstedt, T. P. A. Åkesson, A. V. Akimov, G. L. Alberghi, J. Albert, S. Albrand, M. J. Alconada Verzini, M. Aleksa, I. N. Aleksandrov, C. Alexa, G. Alexander, T. Alexopoulos, M. Alhroob, G. Alimonti, L. Alio, J. Alison, S. P. Alkire, B. M. M. Allbrooke, P. P. Allport, A. Aloisio, A. Alonso, F. Alonso, C. Alpigiani, A. Altheimer, B. Alvarez Gonzalez, D. Álvarez Piqueras, M. G. Alviggi, B. T. Amadio, K. Amako, Y. Amaral Coutinho, C. Amelung, D. Amidei, S. P. Amor Dos Santos, A. Amorim, S. Amoroso, N. Amram, G. Amundsen, C. Anastopoulos, L. S. Ancu, N. Andari, T. Andeen, C. F. Anders, G. Anders, J. K. Anders, K. J. Anderson, A. Andreazza, V. Andrei, S. Angelidakis, I. Angelozzi, P. Anger, A. Angerami, F. Anghinolfi, A. V. Anisenkov, N. Anjos, A. Annovi, M. Antonelli, A. Antonov, J. Antos, F. Anulli, M. Aoki, L. Aperio Bella, G. Arabidze, Y. Arai, J. P. Araque, A. T. H. Arce, F. A. Arduh, J-F. Arguin, S. Argyropoulos, M. Arik, A. J. Armbruster, O. Arnaez, H. Arnold, M. Arratia, O. Arslan, A. Artamonov, G. Artoni, S. Artz, S. Asai, N. Asbah, A. Ashkenazi, B. Åsman, L. Asquith, K. Assamagan, R. Astalos, M. Atkinson, N. B. Atlay, K. Augsten, M. Aurousseau, G. Avolio, B. Axen, M. K. Ayoub, G. Azuelos, M. A. Baak, A. E. Baas, M. J. Baca, H. Bachacou, K. Bachas, M. Backes, M. Backhaus, P. Bagiacchi, P. Bagnaia, Y. Bai, T. Bain, J. T. Baines, O. K. Baker, E. M. Baldin, P. Balek, T. Balestri, F. Balli, W. K. Balunas, E. Banas, Sw. Banerjee, A. A. E. Bannoura, L. Barak, E. L. Barberio, D. Barberis, M. Barbero, T. Barillari, M. Barisonzi, T. Barklow, N. Barlow, S. L. Barnes, B. M. Barnett, R. M. Barnett, Z. Barnovska, A. Baroncelli, G. Barone, A. J. Barr, F. Barreiro, J. Barreiro Guimarães da Costa, R. Bartoldus, A. E. Barton, P. Bartos, A. Basalaev, A. Bassalat, A. Basye, R. L. Bates, S. J. Batista, J. R. Batley, M. Battaglia, M. Bauce, F. Bauer, H. S. Bawa, J. B. Beacham, M. D. Beattie, T. Beau, P. H. Beauchemin, R. Beccherle, P. Bechtle, H. P. Beck, K. Becker, M. Becker, M. Beckingham, C. Becot, A. J. Beddall, A. Beddall, V. A. Bednyakov, C. P. Bee, L. J. Beemster, T. A. Beermann, M. Begel, J. K. Behr, C. Belanger-Champagne, W. H. Bell, G. Bella, L. Bellagamba, A. Bellerive, M. Bellomo, K. Belotskiy, O. Beltramello, O. Benary, D. Benchekroun, M. Bender, K. Bendtz, N. Benekos, Y. Benhammou, E. Benhar Noccioli, J. A. Benitez Garcia, D. P. Benjamin, J. R. Bensinger, S. Bentvelsen, L. Beresford, M. Beretta, D. Berge, E. Bergeaas Kuutmann, N. Berger, F. Berghaus, J. Beringer, C. Bernard, N. R. Bernard, C. Bernius, F. U. Bernlochner, T. Berry, P. Berta, C. Bertella, G. Bertoli, F. Bertolucci, C. Bertsche, D. Bertsche, M. I. Besana, G. J. Besjes, O. Bessidskaia Bylund, M. Bessner, N. Besson, C. Betancourt, S. Bethke, A. J. Bevan, W. Bhimji, R. M. Bianchi, L. Bianchini, M. Bianco, O. Biebel, D. Biedermann, N. V. Biesuz, M. Biglietti, J. Bilbao De Mendizabal, H. Bilokon, M. Bindi, S. Binet, A. Bingul, C. Bini, S. Biondi, D. M. Bjergaard, C. W. Black, J. E. Black, K. M. Black, D. Blackburn, R. E. Blair, J.-B. Blanchard, J. E. Blanco, T. Blazek, I. Bloch, C. Blocker, W. Blum, U. Blumenschein, S. Blunier, G. J. Bobbink, V. S. Bobrovnikov, S. S. Bocchetta, A. Bocci, C. Bock, M. Boehler, J. A. Bogaerts, D. Bogavac, A. G. Bogdanchikov, C. Bohm, V. Boisvert, T. Bold, V. Boldea, A. S. Boldyrev, M. Bomben, M. Bona, M. Boonekamp, A. Borisov, G. Borissov, S. Borroni, J. Bortfeldt, V. Bortolotto, K. Bos, D. Boscherini, M. Bosman, J. Boudreau, J. Bouffard, E. V. Bouhova-Thacker, D. Boumediene, C. Bourdarios, N. Bousson, S. K. Boutle, A. Boveia, J. Boyd, I. R. Boyko, I. Bozic, J. Bracinik, A. Brandt, G. Brandt, O. Brandt, U. Bratzler, B. Brau, J. E. Brau, H. M. Braun, W. D. Breaden Madden, K. Brendlinger, A. J. Brennan, L. Brenner, R. Brenner, S. Bressler, T. M. Bristow, D. Britton, D. Britzger, F. M. Brochu, I. Brock, R. Brock, J. Bronner, G. Brooijmans, T. Brooks, W. K. Brooks, J. Brosamer, E. Brost, P. A. Bruckman de Renstrom, D. Bruncko, R. Bruneliere, A. Bruni, G. Bruni, M. Bruschi, N. Bruscino, L. Bryngemark, T. Buanes, Q. Buat, P. Buchholz, A. G. Buckley, I. A. Budagov, F. Buehrer, L. Bugge, M. K. Bugge, O. Bulekov, D. Bullock, H. Burckhart, S. Burdin, C. D. Burgard, B. Burghgrave, S. Burke, I. Burmeister, E. Busato, D. Büscher, V. Büscher, P. Bussey, J. M. Butler, A. I. Butt, C. M. Buttar, J. M. Butterworth, P. Butti, W. Buttinger, A. Buzatu, A. R. Buzykaev, S. Cabrera Urbán, D. Caforio, V. M. Cairo, O. Cakir, N. Calace, P. Calafiura, A. Calandri, G. Calderini, P. Calfayan, L. P. Caloba, D. Calvet, S. Calvet, R. Camacho Toro, S. Camarda, P. Camarri, D. Cameron, R. Caminal Armadans, S. Campana, M. Campanelli, A. Campoverde, V. Canale, A. Canepa, M. Cano Bret, J. Cantero, R. Cantrill, T. Cao, M. D. M. Capeans Garrido, I. Caprini, M. Caprini, M. Capua, R. Caputo, R. M. Carbone, R. Cardarelli, F. Cardillo, T. Carli, G. Carlino, L. Carminati, S. Caron, E. Carquin, G. D. Carrillo-Montoya, J. R. Carter, J. Carvalho, D. Casadei, M. P. Casado, M. Casolino, D. W. Casper, E. Castaneda-Miranda, A. Castelli, V. Castillo Gimenez, N. F. Castro, P. Catastini, A. Catinaccio, J. R. Catmore, A. Cattai, J. Caudron, V. Cavaliere, D. Cavalli, M. Cavalli-Sforza, V. Cavasinni, F. Ceradini, L. Cerda Alberich, B. C. Cerio, K. Cerny, A. S. Cerqueira, A. Cerri, L. Cerrito, F. Cerutti, M. Cerv, A. Cervelli, S. A. Cetin, A. Chafaq, D. Chakraborty, I. Chalupkova, Y. L. Chan, P. Chang, J. D. Chapman, D. G. Charlton, C. C. Chau, C. A. Chavez Barajas, S. Che, S. Cheatham, A. Chegwidden, S. Chekanov, S. V. Chekulaev, G. A. Chelkov, M. A. Chelstowska, C. Chen, H. Chen, K. Chen, L. Chen, S. Chen, S. Chen, X. Chen, Y. Chen, H. C. Cheng, Y. Cheng, A. Cheplakov, E. Cheremushkina, R. Cherkaoui El Moursli, V. Chernyatin, E. Cheu, L. Chevalier, V. Chiarella, G. Chiarelli, G. Chiodini, A. S. Chisholm, R. T. Chislett, A. Chitan, M. V. Chizhov, K. Choi, S. Chouridou, B. K. B. Chow, V. Christodoulou, D. Chromek-Burckhart, J. Chudoba, A. J. Chuinard, J. J. Chwastowski, L. Chytka, G. Ciapetti, A. K. Ciftci, D. Cinca, V. Cindro, I. A. Cioara, A. Ciocio, F. Cirotto, Z. H. Citron, M. Ciubancan, A. Clark, B. L. Clark, P. J. Clark, R. N. Clarke, C. Clement, Y. Coadou, M. Cobal, A. Coccaro, J. Cochran, L. Coffey, L. Colasurdo, B. Cole, S. Cole, A. P. Colijn, J. Collot, T. Colombo, G. Compostella, P. Conde Muiño, E. Coniavitis, S. H. Connell, I. A. Connelly, V. Consorti, S. Constantinescu, C. Conta, G. Conti, F. Conventi, M. Cooke, B. D. Cooper, A. M. Cooper-Sarkar, T. Cornelissen, M. Corradi, F. Corriveau, A. Corso-Radu, A. Cortes-Gonzalez, G. Cortiana, G. Costa, M. J. Costa, D. Costanzo, D. Côté, G. Cottin, G. Cowan, B. E. Cox, K. Cranmer, S. J. Crawley, G. Cree, S. Crépé-Renaudin, F. Crescioli, W. A. Cribbs, M. Crispin Ortuzar, M. Cristinziani, V. Croft, G. Crosetti, T. Cuhadar Donszelmann, J. Cummings, M. Curatolo, J. Cúth, C. Cuthbert, H. Czirr, P. Czodrowski, S. D’Auria, M. D’Onofrio, M. J. Da Cunha Sargedas De Sousa, C. Da Via, W. Dabrowski, A. Dafinca, T. Dai, O. Dale, F. Dallaire, C. Dallapiccola, M. Dam, J. R. Dandoy, N. P. Dang, A. C. Daniells, M. Danninger, M. Dano Hoffmann, V. Dao, G. Darbo, S. Darmora, J. Dassoulas, A. Dattagupta, W. Davey, C. David, T. Davidek, E. Davies, M. Davies, P. Davison, Y. Davygora, E. Dawe, I. Dawson, R. K. Daya-Ishmukhametova, K. De, R. de Asmundis, A. De Benedetti, S. De Castro, S. De Cecco, N. De Groot, P. de Jong, H. De la Torre, F. De Lorenzi, D. De Pedis, A. De Salvo, U. De Sanctis, A. De Santo, J. B. De Vivie De Regie, W. J. Dearnaley, R. Debbe, C. Debenedetti, D. V. Dedovich, I. Deigaard, J. Del Peso, T. Del Prete, D. Delgove, F. Deliot, C. M. Delitzsch, M. Deliyergiyev, A. Dell’Acqua, L. Dell’Asta, M. Dell’Orso, M. Della Pietra, D. della Volpe, M. Delmastro, P. A. Delsart, C. Deluca, D. A. DeMarco, S. Demers, M. Demichev, A. Demilly, S. P. Denisov, D. Derendarz, J. E. Derkaoui, F. Derue, P. Dervan, K. Desch, C. Deterre, K. Dette, P. O. Deviveiros, A. Dewhurst, S. Dhaliwal, A. Di Ciaccio, L. Di Ciaccio, A. Di Domenico, C. Di Donato, A. Di Girolamo, B. Di Girolamo, A. Di Mattia, B. Di Micco, R. Di Nardo, A. Di Simone, R. Di Sipio, D. Di Valentino, C. Diaconu, M. Diamond, F. A. Dias, M. A. Diaz, E. B. Diehl, J. Dietrich, S. Diglio, A. Dimitrievska, J. Dingfelder, P. Dita, S. Dita, F. Dittus, F. Djama, T. Djobava, J. I. Djuvsland, M. A. B. do Vale, D. Dobos, M. Dobre, C. Doglioni, T. Dohmae, J. Dolejsi, Z. Dolezal, B. A. Dolgoshein, M. Donadelli, S. Donati, P. Dondero, J. Donini, J. Dopke, A. Doria, M. T. Dova, A. T. Doyle, E. Drechsler, M. Dris, Y. Du, E. Dubreuil, E. Duchovni, G. Duckeck, O. A. Ducu, D. Duda, A. Dudarev, L. Duflot, L. Duguid, M. Dührssen, M. Dunford, H. Duran Yildiz, M. Düren, A. Durglishvili, D. Duschinger, B. Dutta, M. Dyndal, C. Eckardt, K. M. Ecker, R. C. Edgar, W. Edson, N. C. Edwards, W. Ehrenfeld, T. Eifert, G. Eigen, K. Einsweiler, T. Ekelof, M. El Kacimi, M. Ellert, S. Elles, F. Ellinghaus, A. A. Elliot, N. Ellis, J. Elmsheuser, M. Elsing, D. Emeliyanov, Y. Enari, O. C. Endner, M. Endo, J. Erdmann, A. Ereditato, G. Ernis, J. Ernst, M. Ernst, S. Errede, E. Ertel, M. Escalier, H. Esch, C. Escobar, B. Esposito, A. I. Etienvre, E. Etzion, H. Evans, A. Ezhilov, L. Fabbri, G. Facini, R. M. Fakhrutdinov, S. Falciano, R. J. Falla, J. Faltova, Y. Fang, M. Fanti, A. Farbin, A. Farilla, T. Farooque, S. Farrell, S. M. Farrington, P. Farthouat, F. Fassi, P. Fassnacht, D. Fassouliotis, M. Faucci Giannelli, A. Favareto, L. Fayard, O. L. Fedin, W. Fedorko, S. Feigl, L. Feligioni, C. Feng, E. J. Feng, H. Feng, A. B. Fenyuk, L. Feremenga, P. Fernandez Martinez, S. Fernandez Perez, J. Ferrando, A. Ferrari, P. Ferrari, R. Ferrari, D. E. Ferreira de Lima, A. Ferrer, D. Ferrere, C. Ferretti, A. Ferretto Parodi, M. Fiascaris, F. Fiedler, A. Filipčič, M. Filipuzzi, F. Filthaut, M. Fincke-Keeler, K. D. Finelli, M. C. N. Fiolhais, L. Fiorini, A. Firan, A. Fischer, C. Fischer, J. Fischer, W. C. Fisher, N. Flaschel, I. Fleck, P. Fleischmann, G. T. Fletcher, G. Fletcher, R. R. M. Fletcher, T. Flick, A. Floderus, L. R. Flores Castillo, M. J. Flowerdew, G. T. Forcolin, A. Formica, A. Forti, D. Fournier, H. Fox, S. Fracchia, P. Francavilla, M. Franchini, D. Francis, L. Franconi, M. Franklin, M. Frate, M. Fraternali, D. Freeborn, S. T. French, S. M. Fressard-Batraneanu, F. Friedrich, D. Froidevaux, J. A. Frost, C. Fukunaga, E. Fullana Torregrosa, B. G. Fulsom, T. Fusayasu, J. Fuster, C. Gabaldon, O. Gabizon, A. Gabrielli, A. Gabrielli, G. P. Gach, S. Gadatsch, S. Gadomski, G. Gagliardi, P. Gagnon, C. Galea, B. Galhardo, E. J. Gallas, B. J. Gallop, P. Gallus, G. Galster, K. K. Gan, J. Gao, Y. Gao, Y. S. Gao, F. M. Garay Walls, C. García, J. E. García Navarro, M. Garcia-Sciveres, R. W. Gardner, N. Garelli, V. Garonne, C. Gatti, A. Gaudiello, G. Gaudio, B. Gaur, L. Gauthier, P. Gauzzi, I. L. Gavrilenko, C. Gay, G. Gaycken, E. N. Gazis, P. Ge, Z. Gecse, C. N. P. Gee, Ch. Geich-Gimbel, M. P. Geisler, C. Gemme, M. H. Genest, C. Geng, S. Gentile, S. George, D. Gerbaudo, A. Gershon, S. Ghasemi, H. Ghazlane, B. Giacobbe, S. Giagu, V. Giangiobbe, P. Giannetti, B. Gibbard, S. M. Gibson, M. Gignac, M. Gilchriese, T. P. S. Gillam, D. Gillberg, G. Gilles, D. M. Gingrich, N. Giokaris, M. P. Giordani, F. M. Giorgi, F. M. Giorgi, P. F. Giraud, P. Giromini, D. Giugni, C. Giuliani, M. Giulini, B. K. Gjelsten, S. Gkaitatzis, I. Gkialas, E. L. Gkougkousis, L. K. Gladilin, C. Glasman, J. Glatzer, P. C. F. Glaysher, A. Glazov, M. Goblirsch-Kolb, J. R. Goddard, J. Godlewski, S. Goldfarb, T. Golling, D. Golubkov, A. Gomes, R. Gonçalo, J. Goncalves Pinto Firmino Da Costa, L. Gonella, S. González de la Hoz, G. Gonzalez Parra, S. Gonzalez-Sevilla, L. Goossens, P. A. Gorbounov, H. A. Gordon, I. Gorelov, B. Gorini, E. Gorini, A. Gorišek, E. Gornicki, A. T. Goshaw, C. Gössling, M. I. Gostkin, D. Goujdami, A. G. Goussiou, N. Govender, E. Gozani, L. Graber, I. Grabowska-Bold, P. O. J. Gradin, P. Grafström, J. Gramling, E. Gramstad, S. Grancagnolo, V. Gratchev, H. M. Gray, E. Graziani, Z. D. Greenwood, C. Grefe, K. Gregersen, I. M. Gregor, P. Grenier, J. Griffiths, A. A. Grillo, K. Grimm, S. Grinstein, Ph. Gris, J.-F. Grivaz, S. Groh, J. P. Grohs, A. Grohsjean, E. Gross, J. Grosse-Knetter, G. C. Grossi, Z. J. Grout, L. Guan, J. Guenther, F. Guescini, D. Guest, O. Gueta, E. Guido, T. Guillemin, S. Guindon, U. Gul, C. Gumpert, J. Guo, Y. Guo, S. Gupta, G. Gustavino, P. Gutierrez, N. G. Gutierrez Ortiz, C. Gutschow, C. Guyot, C. Gwenlan, C. B. Gwilliam, A. Haas, C. Haber, H. K. Hadavand, N. Haddad, P. Haefner, S. Hageböck, Z. Hajduk, H. Hakobyan, M. Haleem, J. Haley, D. Hall, G. Halladjian, G. D. Hallewell, K. Hamacher, P. Hamal, K. Hamano, A. Hamilton, G. N. Hamity, P. G. Hamnett, L. Han, K. Hanagaki, K. Hanawa, M. Hance, B. Haney, P. Hanke, R. Hanna, J. B. Hansen, J. D. Hansen, M. C. Hansen, P. H. Hansen, K. Hara, A. S. Hard, T. Harenberg, F. Hariri, S. Harkusha, R. D. Harrington, P. F. Harrison, F. Hartjes, M. Hasegawa, Y. Hasegawa, A. Hasib, S. Hassani, S. Haug, R. Hauser, L. Hauswald, M. Havranek, C. M. Hawkes, R. J. Hawkings, A. D. Hawkins, T. Hayashi, D. Hayden, C. P. Hays, J. M. Hays, H. S. Hayward, S. J. Haywood, S. J. Head, T. Heck, V. Hedberg, L. Heelan, S. Heim, T. Heim, B. Heinemann, L. Heinrich, J. Hejbal, L. Helary, S. Hellman, C. Helsens, J. Henderson, R. C. W. Henderson, Y. Heng, C. Hengler, S. Henkelmann, A. M. Henriques Correia, S. Henrot-Versille, G. H. Herbert, Y. Hernández Jiménez, G. Herten, R. Hertenberger, L. Hervas, G. G. Hesketh, N. P. Hessey, J. W. Hetherly, R. Hickling, E. Higón-Rodriguez, E. Hill, J. C. Hill, K. H. Hiller, S. J. Hillier, I. Hinchliffe, E. Hines, R. R. Hinman, M. Hirose, D. Hirschbuehl, J. Hobbs, N. Hod, M. C. Hodgkinson, P. Hodgson, A. Hoecker, M. R. Hoeferkamp, F. Hoenig, M. Hohlfeld, D. Hohn, T. R. Holmes, M. Homann, T. M. Hong, B. H. Hooberman, W. H. Hopkins, Y. Horii, A. J. Horton, J-Y. Hostachy, S. Hou, A. Hoummada, J. Howard, J. Howarth, M. Hrabovsky, I. Hristova, J. Hrivnac, T. Hryn’ova, A. Hrynevich, C. Hsu, P. J. Hsu, S.-C. Hsu, D. Hu, Q. Hu, X. Hu, Y. Huang, Z. Hubacek, F. Hubaut, F. Huegging, T. B. Huffman, E. W. Hughes, G. Hughes, M. Huhtinen, T. A. Hülsing, N. Huseynov, J. Huston, J. Huth, G. Iacobucci, G. Iakovidis, I. Ibragimov, L. Iconomidou-Fayard, E. Ideal, Z. Idrissi, P. Iengo, O. Igonkina, T. Iizawa, Y. Ikegami, M. Ikeno, Y. Ilchenko, D. Iliadis, N. Ilic, T. Ince, G. Introzzi, P. Ioannou, M. Iodice, K. Iordanidou, V. Ippolito, A. Irles Quiles, C. Isaksson, M. Ishino, M. Ishitsuka, R. Ishmukhametov, C. Issever, S. Istin, J. M. Iturbe Ponce, R. Iuppa, J. Ivarsson, W. Iwanski, H. Iwasaki, J. M. Izen, V. Izzo, S. Jabbar, B. Jackson, M. Jackson, P. Jackson, M. R. Jaekel, V. Jain, K. B. Jakobi, K. Jakobs, S. Jakobsen, T. Jakoubek, J. Jakubek, D. O. Jamin, D. K. Jana, E. Jansen, R. Jansky, J. Janssen, M. Janus, G. Jarlskog, N. Javadov, T. Javůrek, L. Jeanty, J. Jejelava, G.-Y. Jeng, D. Jennens, P. Jenni, J. Jentzsch, C. Jeske, S. Jézéquel, H. Ji, J. Jia, H. Jiang, Y. Jiang, S. Jiggins, J. Jimenez Pena, S. Jin, A. Jinaru, O. Jinnouchi, M. D. Joergensen, P. Johansson, K. A. Johns, W. J. Johnson, K. Jon-And, G. Jones, R. W. L. Jones, T. J. Jones, J. Jongmanns, P. M. Jorge, K. D. Joshi, J. Jovicevic, X. Ju, A. Juste Rozas, M. Kaci, A. Kaczmarska, M. Kado, H. Kagan, M. Kagan, S. J. Kahn, E. Kajomovitz, C. W. Kalderon, A. Kaluza, S. Kama, A. Kamenshchikov, N. Kanaya, S. Kaneti, V. A. Kantserov, J. Kanzaki, B. Kaplan, L. S. Kaplan, A. Kapliy, D. Kar, K. Karakostas, A. Karamaoun, N. Karastathis, M. J. Kareem, E. Karentzos, M. Karnevskiy, S. N. Karpov, Z. M. Karpova, K. Karthik, V. Kartvelishvili, A. N. Karyukhin, K. Kasahara, L. Kashif, R. D. Kass, A. Kastanas, Y. Kataoka, C. Kato, A. Katre, J. Katzy, K. Kawade, K. Kawagoe, T. Kawamoto, G. Kawamura, S. Kazama, V. F. Kazanin, R. Keeler, R. Kehoe, J. S. Keller, J. J. Kempster, H. Keoshkerian, O. Kepka, B. P. Kerševan, S. Kersten, R. A. Keyes, F. Khalil-zada, H. Khandanyan, A. Khanov, A. G. Kharlamov, T. J. Khoo, V. Khovanskiy, E. Khramov, J. Khubua, S. Kido, H. Y. Kim, S. H. Kim, Y. K. Kim, N. Kimura, O. M. Kind, B. T. King, M. King, S. B. King, J. Kirk, A. E. Kiryunin, T. Kishimoto, D. Kisielewska, F. Kiss, K. Kiuchi, O. Kivernyk, E. Kladiva, M. H. Klein, M. Klein, U. Klein, K. Kleinknecht, P. Klimek, A. Klimentov, R. Klingenberg, J. A. Klinger, T. Klioutchnikova, E.-E. Kluge, P. Kluit, S. Kluth, J. Knapik, E. Kneringer, E. B. F. G. Knoops, A. Knue, A. Kobayashi, D. Kobayashi, T. Kobayashi, M. Kobel, M. Kocian, P. Kodys, T. Koffas, E. Koffeman, L. A. Kogan, S. Kohlmann, Z. Kohout, T. Kohriki, T. Koi, H. Kolanoski, M. Kolb, I. Koletsou, A. A. Komar, Y. Komori, T. Kondo, N. Kondrashova, K. Köneke, A. C. König, T. Kono, R. Konoplich, N. Konstantinidis, R. Kopeliansky, S. Koperny, L. Köpke, A. K. Kopp, K. Korcyl, K. Kordas, A. Korn, A. A. Korol, I. Korolkov, E. V. Korolkova, O. Kortner, S. Kortner, T. Kosek, V. V. Kostyukhin, V. M. Kotov, A. Kotwal, A. Kourkoumeli-Charalampidi, C. Kourkoumelis, V. Kouskoura, A. Koutsman, R. Kowalewski, T. Z. Kowalski, W. Kozanecki, A. S. Kozhin, V. A. Kramarenko, G. Kramberger, D. Krasnopevtsev, M. W. Krasny, A. Krasznahorkay, J. K. Kraus, A. Kravchenko, S. Kreiss, M. Kretz, J. Kretzschmar, K. Kreutzfeldt, P. Krieger, K. Krizka, K. Kroeninger, H. Kroha, J. Kroll, J. Kroseberg, J. Krstic, U. Kruchonak, H. Krüger, N. Krumnack, A. Kruse, M. C. Kruse, M. Kruskal, T. Kubota, H. Kucuk, S. Kuday, S. Kuehn, A. Kugel, F. Kuger, A. Kuhl, T. Kuhl, V. Kukhtin, R. Kukla, Y. Kulchitsky, S. Kuleshov, M. Kuna, T. Kunigo, A. Kupco, H. Kurashige, Y. A. Kurochkin, V. Kus, E. S. Kuwertz, M. Kuze, J. Kvita, T. Kwan, D. Kyriazopoulos, A. La Rosa, J. L. La Rosa Navarro, L. La Rotonda, C. Lacasta, F. Lacava, J. Lacey, H. Lacker, D. Lacour, V. R. Lacuesta, E. Ladygin, R. Lafaye, B. Laforge, T. Lagouri, S. Lai, L. Lambourne, S. Lammers, C. L. Lampen, W. Lampl, E. Lançon, U. Landgraf, M. P. J. Landon, V. S. Lang, J. C. Lange, A. J. Lankford, F. Lanni, K. Lantzsch, A. Lanza, S. Laplace, C. Lapoire, J. F. Laporte, T. Lari, F. Lasagni Manghi, M. Lassnig, P. Laurelli, W. Lavrijsen, A. T. Law, P. Laycock, T. Lazovich, O. Le Dortz, E. Le Guirriec, E. Le Menedeu, M. LeBlanc, T. LeCompte, F. Ledroit-Guillon, C. A. Lee, S. C. Lee, L. Lee, G. Lefebvre, M. Lefebvre, F. Legger, C. Leggett, A. Lehan, G. Lehmann Miotto, X. Lei, W. A. Leight, A. Leisos, A. G. Leister, M. A. L. Leite, R. Leitner, D. Lellouch, B. Lemmer, K. J. C. Leney, T. Lenz, B. Lenzi, R. Leone, S. Leone, C. Leonidopoulos, S. Leontsinis, C. Leroy, C. G. Lester, M. Levchenko, J. Levêque, D. Levin, L. J. Levinson, M. Levy, A. Lewis, A. M. Leyko, M. Leyton, B. Li, H. Li, H. L. Li, L. Li, L. Li, S. Li, X. Li, Y. Li, Z. Liang, H. Liao, B. Liberti, A. Liblong, P. Lichard, K. Lie, J. Liebal, W. Liebig, C. Limbach, A. Limosani, S. C. Lin, T. H. Lin, F. Linde, B. E. Lindquist, J. T. Linnemann, E. Lipeles, A. Lipniacka, M. Lisovyi, T. M. Liss, D. Lissauer, A. Lister, A. M. Litke, B. Liu, D. Liu, H. Liu, J. Liu, J. B. Liu, K. Liu, L. Liu, M. Liu, M. Liu, Y. Liu, M. Livan, A. Lleres, J. Llorente Merino, S. L. Lloyd, F. Lo Sterzo, E. Lobodzinska, P. Loch, W. S. Lockman, F. K. Loebinger, A. E. Loevschall-Jensen, K. M. Loew, A. Loginov, T. Lohse, K. Lohwasser, M. Lokajicek, B. A. Long, J. D. Long, R. E. Long, K. A. Looper, L. Lopes, D. Lopez Mateos, B. Lopez Paredes, I. Lopez Paz, J. Lorenz, N. Lorenzo Martinez, M. Losada, P. J. Lösel, X. Lou, A. Lounis, J. Love, P. A. Love, H. Lu, N. Lu, H. J. Lubatti, C. Luci, A. Lucotte, C. Luedtke, F. Luehring, W. Lukas, L. Luminari, O. Lundberg, B. Lund-Jensen, D. Lynn, R. Lysak, E. Lytken, H. Ma, L. L. Ma, G. Maccarrone, A. Macchiolo, C. M. Macdonald, B. Maček, J. Machado Miguens, D. Macina, D. Madaffari, R. Madar, H. J. Maddocks, W. F. Mader, A. Madsen, J. Maeda, S. Maeland, T. Maeno, A. Maevskiy, E. Magradze, K. Mahboubi, J. Mahlstedt, C. Maiani, C. Maidantchik, A. A. Maier, T. Maier, A. Maio, S. Majewski, Y. Makida, N. Makovec, B. Malaescu, Pa. Malecki, V. P. Maleev, F. Malek, U. Mallik, D. Malon, C. Malone, S. Maltezos, V. M. Malyshev, S. Malyukov, J. Mamuzic, G. Mancini, B. Mandelli, L. Mandelli, I. Mandić, R. Mandrysch, J. Maneira, L. Manhaes de Andrade Filho, J. Manjarres Ramos, A. Mann, A. Manousakis-Katsikakis, B. Mansoulie, R. Mantifel, M. Mantoani, L. Mapelli, L. March, G. Marchiori, M. Marcisovsky, C. P. Marino, M. Marjanovic, D. E. Marley, F. Marroquim, S. P. Marsden, Z. Marshall, L. F. Marti, S. Marti-Garcia, B. Martin, T. A. Martin, V. J. Martin, B. Martin dit Latour, M. Martinez, S. Martin-Haugh, V. S. Martoiu, A. C. Martyniuk, M. Marx, F. Marzano, A. Marzin, L. Masetti, T. Mashimo, R. Mashinistov, J. Masik, A. L. Maslennikov, I. Massa, L. Massa, P. Mastrandrea, A. Mastroberardino, T. Masubuchi, P. Mättig, J. Mattmann, J. Maurer, S. J. Maxfield, D. A. Maximov, R. Mazini, S. M. Mazza, G. Mc Goldrick, S. P. Mc Kee, A. McCarn, R. L. McCarthy, T. G. McCarthy, N. A. McCubbin, K. W. McFarlane, J. A. Mcfayden, G. Mchedlidze, S. J. McMahon, R. A. McPherson, M. Medinnis, S. Meehan, S. Mehlhase, A. Mehta, K. Meier, C. Meineck, B. Meirose, B. R. Mellado Garcia, F. Meloni, A. Mengarelli, S. Menke, E. Meoni, K. M. Mercurio, S. Mergelmeyer, P. Mermod, L. Merola, C. Meroni, F. S. Merritt, A. Messina, J. Metcalfe, A. S. Mete, C. Meyer, C. Meyer, J-P. Meyer, J. Meyer, H. Meyer Zu Theenhausen, R. P. Middleton, S. Miglioranzi, L. Mijović, G. Mikenberg, M. Mikestikova, M. Mikuž, M. Milesi, A. Milic, D. W. Miller, C. Mills, A. Milov, D. A. Milstead, A. A. Minaenko, Y. Minami, I. A. Minashvili, A. I. Mincer, B. Mindur, M. Mineev, Y. Ming, L. M. Mir, K. P. Mistry, T. Mitani, J. Mitrevski, V. A. Mitsou, A. Miucci, P. S. Miyagawa, J. U. Mjörnmark, T. Moa, K. Mochizuki, S. Mohapatra, W. Mohr, S. Molander, R. Moles-Valls, R. Monden, M. C. Mondragon, K. Mönig, C. Monini, J. Monk, E. Monnier, A. Montalbano, J. Montejo Berlingen, F. Monticelli, S. Monzani, R. W. Moore, N. Morange, D. Moreno, M. Moreno Llácer, P. Morettini, D. Mori, T. Mori, M. Morii, M. Morinaga, V. Morisbak, S. Moritz, A. K. Morley, G. Mornacchi, J. D. Morris, S. S. Mortensen, A. Morton, L. Morvaj, M. Mosidze, J. Moss, K. Motohashi, R. Mount, E. Mountricha, S. V. Mouraviev, E. J. W. Moyse, S. Muanza, R. D. Mudd, F. Mueller, J. Mueller, R. S. P. Mueller, T. Mueller, D. Muenstermann, P. Mullen, G. A. Mullier, F. J. Munoz Sanchez, J. A. Murillo Quijada, W. J. Murray, H. Musheghyan, E. Musto, A. G. Myagkov, M. Myska, B. P. Nachman, O. Nackenhorst, J. Nadal, K. Nagai, R. Nagai, Y. Nagai, K. Nagano, A. Nagarkar, Y. Nagasaka, K. Nagata, M. Nagel, E. Nagy, A. M. Nairz, Y. Nakahama, K. Nakamura, T. Nakamura, I. Nakano, H. Namasivayam, R. F. Naranjo Garcia, R. Narayan, D. I. Narrias Villar, T. Naumann, G. Navarro, R. Nayyar, H. A. Neal, P. Yu. Nechaeva, T. J. Neep, P. D. Nef, A. Negri, M. Negrini, S. Nektarijevic, C. Nellist, A. Nelson, S. Nemecek, P. Nemethy, A. A. Nepomuceno, M. Nessi, M. S. Neubauer, M. Neumann, R. M. Neves, P. Nevski, P. R. Newman, D. H. Nguyen, R. B. Nickerson, R. Nicolaidou, B. Nicquevert, J. Nielsen, N. Nikiforou, A. Nikiforov, V. Nikolaenko, I. Nikolic-Audit, K. Nikolopoulos, J. K. Nilsen, P. Nilsson, Y. Ninomiya, A. Nisati, R. Nisius, T. Nobe, L. Nodulman, M. Nomachi, I. Nomidis, T. Nooney, S. Norberg, M. Nordberg, O. Novgorodova, S. Nowak, M. Nozaki, L. Nozka, K. Ntekas, T. Nunnemann, E. Nurse, F. Nuti, F. O’grady, D. C. O’Neil, V. O’Shea, F. G. Oakham, H. Oberlack, T. Obermann, J. Ocariz, A. Ochi, I. Ochoa, J. P. Ochoa-Ricoux, S. Oda, S. Odaka, H. Ogren, A. Oh, S. H. Oh, C. C. Ohm, H. Ohman, H. Oide, W. Okamura, H. Okawa, Y. Okumura, T. Okuyama, A. Olariu, S. A. Olivares Pino, D. Oliveira Damazio, A. Olszewski, J. Olszowska, A. Onofre, K. Onogi, P. U. E. Onyisi, C. J. Oram, M. J. Oreglia, Y. Oren, D. Orestano, N. Orlando, C. Oropeza Barrera, R. S. Orr, B. Osculati, R. Ospanov, G. Otero y Garzon, H. Otono, M. Ouchrif, F. Ould-Saada, A. Ouraou, K. P. Oussoren, Q. Ouyang, A. Ovcharova, M. Owen, R. E. Owen, V. E. Ozcan, N. Ozturk, K. Pachal, A. Pacheco Pages, C. Padilla Aranda, M. Pagáčová, S. Pagan Griso, E. Paganis, F. Paige, P. Pais, K. Pajchel, G. Palacino, S. Palestini, M. Palka, D. Pallin, A. Palma, Y. B. Pan, E. St. Panagiotopoulou, C. E. Pandini, J. G. Panduro Vazquez, P. Pani, S. Panitkin, D. Pantea, L. Paolozzi, Th. D. Papadopoulou, K. Papageorgiou, A. Paramonov, D. Paredes Hernandez, M. A. Parker, K. A. Parker, F. Parodi, J. A. Parsons, U. Parzefall, E. Pasqualucci, S. Passaggio, F. Pastore, Fr. Pastore, G. Pásztor, S. Pataraia, N. D. Patel, J. R. Pater, T. Pauly, J. Pearce, B. Pearson, L. E. Pedersen, M. Pedersen, S. Pedraza Lopez, R. Pedro, S. V. Peleganchuk, D. Pelikan, O. Penc, C. Peng, H. Peng, B. Penning, J. Penwell, D. V. Perepelitsa, E. Perez Codina, M. T. Pérez García-Estañ, L. Perini, H. Pernegger, S. Perrella, R. Peschke, V. D. Peshekhonov, K. Peters, R. F. Y. Peters, B. A. Petersen, T. C. Petersen, E. Petit, A. Petridis, C. Petridou, P. Petroff, E. Petrolo, F. Petrucci, N. E. Pettersson, R. Pezoa, P. W. Phillips, G. Piacquadio, E. Pianori, A. Picazio, E. Piccaro, M. Piccinini, M. A. Pickering, R. Piegaia, D. T. Pignotti, J. E. Pilcher, A. D. Pilkington, A. W. J. Pin, J. Pina, M. Pinamonti, J. L. Pinfold, A. Pingel, S. Pires, H. Pirumov, M. Pitt, C. Pizio, L. Plazak, M.-A. Pleier, V. Pleskot, E. Plotnikova, P. Plucinski, D. Pluth, R. Poettgen, L. Poggioli, D. Pohl, G. Polesello, A. Poley, A. Policicchio, R. Polifka, A. Polini, C. S. Pollard, V. Polychronakos, K. Pommès, L. Pontecorvo, B. G. Pope, G. A. Popeneciu, D. S. Popovic, A. Poppleton, S. Pospisil, K. Potamianos, I. N. Potrap, C. J. Potter, C. T. Potter, G. Poulard, J. Poveda, V. Pozdnyakov, M. E. Pozo Astigarraga, P. Pralavorio, A. Pranko, S. Prasad, S. Prell, D. Price, L. E. Price, M. Primavera, S. Prince, M. Proissl, K. Prokofiev, F. Prokoshin, E. Protopapadaki, S. Protopopescu, J. Proudfoot, M. Przybycien, E. Ptacek, D. Puddu, E. Pueschel, D. Puldon, M. Purohit, P. Puzo, J. Qian, G. Qin, Y. Qin, A. Quadt, D. R. Quarrie, W. B. Quayle, M. Queitsch-Maitland, D. Quilty, S. Raddum, V. Radeka, V. Radescu, S. K. Radhakrishnan, P. Radloff, P. Rados, F. Ragusa, G. Rahal, S. Rajagopalan, M. Rammensee, C. Rangel-Smith, F. Rauscher, S. Rave, T. Ravenscroft, M. Raymond, A. L. Read, N. P. Readioff, D. M. Rebuzzi, A. Redelbach, G. Redlinger, R. Reece, K. Reeves, L. Rehnisch, J. Reichert, H. Reisin, C. Rembser, H. Ren, A. Renaud, M. Rescigno, S. Resconi, O. L. Rezanova, P. Reznicek, R. Rezvani, R. Richter, S. Richter, E. Richter-Was, O. Ricken, M. Ridel, P. Rieck, C. J. Riegel, J. Rieger, O. Rifki, M. Rijssenbeek, A. Rimoldi, L. Rinaldi, B. Ristić, E. Ritsch, I. Riu, F. Rizatdinova, E. Rizvi, S. H. Robertson, A. Robichaud-Veronneau, D. Robinson, J. E. M. Robinson, A. Robson, C. Roda, S. Roe, O. Røhne, A. Romaniouk, M. Romano, S. M. Romano Saez, E. Romero Adam, N. Rompotis, M. Ronzani, L. Roos, E. Ros, S. Rosati, K. Rosbach, P. Rose, O. Rosenthal, V. Rossetti, E. Rossi, L. P. Rossi, J. H. N. Rosten, R. Rosten, M. Rotaru, I. Roth, J. Rothberg, D. Rousseau, C. R. Royon, A. Rozanov, Y. Rozen, X. Ruan, F. Rubbo, I. Rubinskiy, V. I. Rud, C. Rudolph, M. S. Rudolph, F. Rühr, A. Ruiz-Martinez, Z. Rurikova, N. A. Rusakovich, A. Ruschke, H. L. Russell, J. P. Rutherfoord, N. Ruthmann, Y. F. Ryabov, M. Rybar, G. Rybkin, N. C. Ryder, A. Ryzhov, A. F. Saavedra, G. Sabato, S. Sacerdoti, A. Saddique, H. F-W. Sadrozinski, R. Sadykov, F. Safai Tehrani, P. Saha, M. Sahinsoy, M. Saimpert, T. Saito, H. Sakamoto, Y. Sakurai, G. Salamanna, A. Salamon, J. E. Salazar Loyola, M. Saleem, D. Salek, P. H. Sales De Bruin, D. Salihagic, A. Salnikov, J. Salt, D. Salvatore, F. Salvatore, A. Salvucci, A. Salzburger, D. Sammel, D. Sampsonidis, A. Sanchez, J. Sánchez, V. Sanchez Martinez, H. Sandaker, R. L. Sandbach, H. G. Sander, M. P. Sanders, M. Sandhoff, C. Sandoval, R. Sandstroem, D. P. C. Sankey, M. Sannino, A. Sansoni, C. Santoni, R. Santonico, H. Santos, I. Santoyo Castillo, K. Sapp, A. Sapronov, J. G. Saraiva, B. Sarrazin, O. Sasaki, Y. Sasaki, K. Sato, G. Sauvage, E. Sauvan, G. Savage, P. Savard, C. Sawyer, L. Sawyer, J. Saxon, C. Sbarra, A. Sbrizzi, T. Scanlon, D. A. Scannicchio, M. Scarcella, V. Scarfone, J. Schaarschmidt, P. Schacht, D. Schaefer, R. Schaefer, J. Schaeffer, S. Schaepe, S. Schaetzel, U. Schäfer, A. C. Schaffer, D. Schaile, R. D. Schamberger, V. Scharf, V. A. Schegelsky, D. Scheirich, M. Schernau, C. Schiavi, C. Schillo, M. Schioppa, S. Schlenker, K. Schmieden, C. Schmitt, S. Schmitt, S. Schmitt, S. Schmitz, B. Schneider, Y. J. Schnellbach, U. Schnoor, L. Schoeffel, A. Schoening, B. D. Schoenrock, E. Schopf, A. L. S. Schorlemmer, M. Schott, D. Schouten, J. Schovancova, S. Schramm, M. Schreyer, N. Schuh, M. J. Schultens, H.-C. Schultz-Coulon, H. Schulz, M. Schumacher, B. A. Schumm, Ph. Schune, C. Schwanenberger, A. Schwartzman, T. A. Schwarz, Ph. Schwegler, H. Schweiger, Ph. Schwemling, R. Schwienhorst, J. Schwindling, T. Schwindt, E. Scifo, G. Sciolla, F. Scuri, F. Scutti, J. Searcy, G. Sedov, E. Sedykh, P. Seema, S. C. Seidel, A. Seiden, F. Seifert, J. M. Seixas, G. Sekhniaidze, K. Sekhon, S. J. Sekula, D. M. Seliverstov, N. Semprini-Cesari, C. Serfon, L. Serin, L. Serkin, T. Serre, M. Sessa, R. Seuster, H. Severini, T. Sfiligoj, F. Sforza, A. Sfyrla, E. Shabalina, M. Shamim, L. Y. Shan, R. Shang, J. T. Shank, M. Shapiro, P. B. Shatalov, K. Shaw, S. M. Shaw, A. Shcherbakova, C. Y. Shehu, P. Sherwood, L. Shi, S. Shimizu, C. O. Shimmin, M. Shimojima, M. Shiyakova, A. Shmeleva, D. Shoaleh Saadi, M. J. Shochet, S. Shojaii, S. Shrestha, E. Shulga, M. A. Shupe, P. Sicho, P. E. Sidebo, O. Sidiropoulou, D. Sidorov, A. Sidoti, F. Siegert, Dj. Sijacki, J. Silva, Y. Silver, S. B. Silverstein, V. Simak, O. Simard, Lj. Simic, S. Simion, E. Simioni, B. Simmons, D. Simon, M. Simon, P. Sinervo, N. B. Sinev, M. Sioli, G. Siragusa, S. Yu. Sivoklokov, J. Sjölin, T. B. Sjursen, M. B. Skinner, H. P. Skottowe, P. Skubic, M. Slater, T. Slavicek, M. Slawinska, K. Sliwa, V. Smakhtin, B. H. Smart, L. Smestad, S. Yu. Smirnov, Y. Smirnov, L. N. Smirnova, O. Smirnova, M. N. K. Smith, R. W. Smith, M. Smizanska, K. Smolek, A. A. Snesarev, G. Snidero, S. Snyder, R. Sobie, F. Socher, A. Soffer, D. A. Soh, G. Sokhrannyi, C. A. Solans, M. Solar, J. Solc, E. Yu. Soldatov, U. Soldevila, A. A. Solodkov, A. Soloshenko, O. V. Solovyanov, V. Solovyev, P. Sommer, H. Y. Song, N. Soni, A. Sood, A. Sopczak, B. Sopko, V. Sopko, V. Sorin, D. Sosa, M. Sosebee, C. L. Sotiropoulou, R. Soualah, A. M. Soukharev, D. South, B. C. Sowden, S. Spagnolo, M. Spalla, M. Spangenberg, F. Spanò, W. R. Spearman, D. Sperlich, F. Spettel, R. Spighi, G. Spigo, L. A. Spiller, M. Spousta, R. D. St. Denis, A. Stabile, S. Staerz, J. Stahlman, R. Stamen, S. Stamm, E. Stanecka, R. W. Stanek, C. Stanescu, M. Stanescu-Bellu, M. M. Stanitzki, S. Stapnes, E. A. Starchenko, J. Stark, P. Staroba, P. Starovoitov, R. Staszewski, P. Steinberg, B. Stelzer, H. J. Stelzer, O. Stelzer-Chilton, H. Stenzel, G. A. Stewart, J. A. Stillings, M. C. Stockton, M. Stoebe, G. Stoicea, P. Stolte, S. Stonjek, A. R. Stradling, A. Straessner, M. E. Stramaglia, J. Strandberg, S. Strandberg, A. Strandlie, E. Strauss, M. Strauss, P. Strizenec, R. Ströhmer, D. M. Strom, R. Stroynowski, A. Strubig, S. A. Stucci, B. Stugu, N. A. Styles, D. Su, J. Su, R. Subramaniam, A. Succurro, S. Suchek, Y. Sugaya, M. Suk, V. V. Sulin, S. Sultansoy, T. Sumida, S. Sun, X. Sun, J. E. Sundermann, K. Suruliz, G. Susinno, M. R. Sutton, S. Suzuki, M. Svatos, M. Swiatlowski, I. Sykora, T. Sykora, D. Ta, C. Taccini, K. Tackmann, J. Taenzer, A. Taffard, R. Tafirout, N. Taiblum, H. Takai, R. Takashima, H. Takeda, T. Takeshita, Y. Takubo, M. Talby, A. A. Talyshev, J. Y. C. Tam, K. G. Tan, J. Tanaka, R. Tanaka, S. Tanaka, B. B. Tannenwald, S. Tapia Araya, S. Tapprogge, S. Tarem, F. Tarrade, G. F. Tartarelli, P. Tas, M. Tasevsky, T. Tashiro, E. Tassi, A. Tavares Delgado, Y. Tayalati, A. C. Taylor, F. E. Taylor, G. N. Taylor, P. T. E. Taylor, W. Taylor, F. A. Teischinger, P. Teixeira-Dias, K. K. Temming, D. Temple, H. Ten Kate, P. K. Teng, J. J. Teoh, F. Tepel, S. Terada, K. Terashi, J. Terron, S. Terzo, M. Testa, R. J. Teuscher, T. Theveneaux-Pelzer, J. P. Thomas, J. Thomas-Wilsker, E. N. Thompson, P. D. Thompson, R. J. Thompson, A. S. Thompson, L. A. Thomsen, E. Thomson, M. Thomson, R. P. Thun, M. J. Tibbetts, R. E. Ticse Torres, V. O. Tikhomirov, Yu. A. Tikhonov, S. Timoshenko, E. Tiouchichine, P. Tipton, S. Tisserant, K. Todome, T. Todorov, S. Todorova-Nova, J. Tojo, S. Tokár, K. Tokushuku, K. Tollefson, E. Tolley, L. Tomlinson, M. Tomoto, L. Tompkins, K. Toms, E. Torrence, H. Torres, E. Torró Pastor, J. Toth, F. Touchard, D. R. Tovey, T. Trefzger, L. Tremblet, A. Tricoli, I. M. Trigger, S. Trincaz-Duvoid, M. F. Tripiana, W. Trischuk, B. Trocmé, C. Troncon, M. Trottier-McDonald, M. Trovatelli, L. Truong, M. Trzebinski, A. Trzupek, C. Tsarouchas, J. C-L. Tseng, P. V. Tsiareshka, D. Tsionou, G. Tsipolitis, N. Tsirintanis, S. Tsiskaridze, V. Tsiskaridze, E. G. Tskhadadze, K. M. Tsui, I. I. Tsukerman, V. Tsulaia, S. Tsuno, D. Tsybychev, A. Tudorache, V. Tudorache, A. N. Tuna, S. A. Tupputi, S. Turchikhin, D. Turecek, R. Turra, A. J. Turvey, P. M. Tuts, A. Tykhonov, M. Tylmad, M. Tyndel, I. Ueda, R. Ueno, M. Ughetto, F. Ukegawa, G. Unal, A. Undrus, G. Unel, F. C. Ungaro, Y. Unno, C. Unverdorben, J. Urban, P. Urquijo, P. Urrejola, G. Usai, A. Usanova, L. Vacavant, V. Vacek, B. Vachon, C. Valderanis, N. Valencic, S. Valentinetti, A. Valero, L. Valery, S. Valkar, S. Vallecorsa, J. A. Valls Ferrer, W. Van Den Wollenberg, P. C. Van Der Deijl, R. van der Geer, H. van der Graaf, N. van Eldik, P. van Gemmeren, J. Van Nieuwkoop, I. van Vulpen, M. C. van Woerden, M. Vanadia, W. Vandelli, R. Vanguri, A. Vaniachine, F. Vannucci, G. Vardanyan, R. Vari, E. W. Varnes, T. Varol, D. Varouchas, A. Vartapetian, K. E. Varvell, F. Vazeille, T. Vazquez Schroeder, J. Veatch, L. M. Veloce, F. Veloso, T. Velz, S. Veneziano, A. Ventura, D. Ventura, M. Venturi, N. Venturi, A. Venturini, V. Vercesi, M. Verducci, W. Verkerke, J. C. Vermeulen, A. Vest, M. C. Vetterli, O. Viazlo, I. Vichou, T. Vickey, O. E. Vickey Boeriu, G. H. A. Viehhauser, S. Viel, R. Vigne, M. Villa, M. Villaplana Perez, E. Vilucchi, M. G. Vincter, V. B. Vinogradov, I. Vivarelli, S. Vlachos, D. Vladoiu, M. Vlasak, M. Vogel, P. Vokac, G. Volpi, M. Volpi, H. von der Schmitt, H. von Radziewski, E. von Toerne, V. Vorobel, K. Vorobev, M. Vos, R. Voss, J. H. Vossebeld, N. Vranjes, M. Vranjes Milosavljevic, V. Vrba, M. Vreeswijk, R. Vuillermet, I. Vukotic, Z. Vykydal, P. Wagner, W. Wagner, H. Wahlberg, S. Wahrmund, J. Wakabayashi, J. Walder, R. Walker, W. Walkowiak, C. Wang, F. Wang, H. Wang, H. Wang, J. Wang, J. Wang, K. Wang, R. Wang, S. M. Wang, T. Wang, T. Wang, X. Wang, C. Wanotayaroj, A. Warburton, C. P. Ward, D. R. Wardrope, A. Washbrook, C. Wasicki, P. M. Watkins, A. T. Watson, I. J. Watson, M. F. Watson, G. Watts, S. Watts, B. M. Waugh, S. Webb, M. S. Weber, S. W. Weber, J. S. Webster, A. R. Weidberg, B. Weinert, J. Weingarten, C. Weiser, H. Weits, P. S. Wells, T. Wenaus, T. Wengler, S. Wenig, N. Wermes, M. Werner, P. Werner, M. Wessels, J. Wetter, K. Whalen, A. M. Wharton, A. White, M. J. White, R. White, S. White, D. Whiteson, F. J. Wickens, W. Wiedenmann, M. Wielers, P. Wienemann, C. Wiglesworth, L. A. M. Wiik-Fuchs, A. Wildauer, H. G. Wilkens, H. H. Williams, S. Williams, C. Willis, S. Willocq, A. Wilson, J. A. Wilson, I. Wingerter-Seez, F. Winklmeier, B. T. Winter, M. Wittgen, J. Wittkowski, S. J. Wollstadt, M. W. Wolter, H. Wolters, B. K. Wosiek, J. Wotschack, M. J. Woudstra, K. W. Wozniak, M. Wu, M. Wu, S. L. Wu, X. Wu, Y. Wu, T. R. Wyatt, B. M. Wynne, S. Xella, D. Xu, L. Xu, B. Yabsley, S. Yacoob, R. Yakabe, M. Yamada, D. Yamaguchi, Y. Yamaguchi, A. Yamamoto, S. Yamamoto, T. Yamanaka, K. Yamauchi, Y. Yamazaki, Z. Yan, H. Yang, H. Yang, Y. Yang, W-M. Yao, Y. C. Yap, Y. Yasu, E. Yatsenko, K. H. Yau Wong, J. Ye, S. Ye, I. Yeletskikh, A. L. Yen, E. Yildirim, K. Yorita, R. Yoshida, K. Yoshihara, C. Young, C. J. S. Young, S. Youssef, D. R. Yu, J. Yu, J. M. Yu, J. Yu, L. Yuan, S. P. Y. Yuen, A. Yurkewicz, I. Yusuff, B. Zabinski, R. Zaidan, A. M. Zaitsev, J. Zalieckas, A. Zaman, S. Zambito, L. Zanello, D. Zanzi, C. Zeitnitz, M. Zeman, A. Zemla, J. C. Zeng, Q. Zeng, K. Zengel, O. Zenin, T. Ženiš, D. Zerwas, D. Zhang, F. Zhang, G. Zhang, H. Zhang, J. Zhang, L. Zhang, R. Zhang, X. Zhang, Z. Zhang, X. Zhao, Y. Zhao, Z. Zhao, A. Zhemchugov, J. Zhong, B. Zhou, C. Zhou, L. Zhou, L. Zhou, M. Zhou, N. Zhou, C. G. Zhu, H. Zhu, J. Zhu, Y. Zhu, X. Zhuang, K. Zhukov, A. Zibell, D. Zieminska, N. I. Zimine, C. Zimmermann, S. Zimmermann, Z. Zinonos, M. Zinser, M. Ziolkowski, L. Živković, G. Zobernig, A. Zoccoli, M. zur Nedden, G. Zurzolo, L. Zwalinski

**Affiliations:** 1 Department of Physics, University of Adelaide, Adelaide, Australia; 2 Physics Department, SUNY Albany, Albany, NY USA; 3 Department of Physics, University of Alberta, Edmonton, AB Canada; 4 Department of Physics, Ankara University, Ankara, Turkey; 5 Istanbul Aydin University, Istanbul, Turkey; 6 Division of Physics, TOBB University of Economics and Technology, Ankara, Turkey; 7 LAPP, CNRS/IN2P3 and Université Savoie Mont Blanc, Annecy-le-Vieux, France; 8 High Energy Physics Division, Argonne National Laboratory, Argonne, IL USA; 9 Department of Physics, University of Arizona, Tucson, AZ USA; 10 Department of Physics, The University of Texas at Arlington, Arlington, TX USA; 11 Physics Department, University of Athens, Athens, Greece; 12 Physics Department, National Technical University of Athens, Zografou, Greece; 13 Institute of Physics, Azerbaijan Academy of Sciences, Baku, Azerbaijan; 14 Institut de Física d’Altes Energies (IFAE), The Barcelona Institute of Science and Technology, Barcelona, Spain; 15 Institute of Physics, University of Belgrade, Belgrade, Serbia; 16 Department for Physics and Technology, University of Bergen, Bergen, Norway; 17 Physics Division, Lawrence Berkeley National Laboratory and University of California, Berkeley, CA USA; 18 Department of Physics, Humboldt University, Berlin, Germany; 19 Albert Einstein Center for Fundamental Physics and Laboratory for High Energy Physics, University of Bern, Bern, Switzerland; 20 School of Physics and Astronomy, University of Birmingham, Birmingham, UK; 21 Department of Physics, Bogazici University, Istanbul, Turkey; 22 Department of Physics Engineering, Gaziantep University, Gaziantep, Turkey; 23 Department of Physics, Dogus University, Istanbul, Turkey; 24 INFN Sezione di Bologna, Bologna, Italy; 25 Dipartimento di Fisica e Astronomia, Università di Bologna, Bologna, Italy; 26 Physikalisches Institut, University of Bonn, Bonn, Germany; 27 Department of Physics, Boston University, Boston, MA USA; 28 Department of Physics, Brandeis University, Waltham, MA USA; 29 Universidade Federal do Rio De Janeiro COPPE/EE/IF, Rio de Janeiro, Brazil; 30 Electrical Circuits Department, Federal University of Juiz de Fora (UFJF), Juiz de Fora, Brazil; 31 Federal University of Sao Joao del Rei (UFSJ), Sao Joao del Rei, Brazil; 32 Instituto de Fisica, Universidade de Sao Paulo, São Paulo, Brazil; 33 Physics Department, Brookhaven National Laboratory, Upton, NY USA; 34 Transilvania University of Brasov, Brasov, Romania; 35 National Institute of Physics and Nuclear Engineering, Bucharest, Romania; 36 Physics Department, National Institute for Research and Development of Isotopic and Molecular Technologies, Cluj Napoca, Romania; 37 University Politehnica Bucharest, Bucharest, Romania; 38 West University in Timisoara, Timisoara, Romania; 39 Departamento de Física, Universidad de Buenos Aires, Buenos Aires, Argentina; 40 Cavendish Laboratory, University of Cambridge, Cambridge, UK; 41 Department of Physics, Carleton University, Ottawa, ON Canada; 42 CERN, Geneva, Switzerland; 43 Enrico Fermi Institute, University of Chicago, Chicago, IL USA; 44 Departamento de Física, Pontificia Universidad Católica de Chile, Santiago, Chile; 45 Departamento de Física, Universidad Técnica Federico Santa María, Valparaiso, Chile; 46 Institute of High Energy Physics, Chinese Academy of Sciences, Beijing, China; 47 Department of Modern Physics, University of Science and Technology of China, Hefei, Anhui China; 48 Department of Physics, Nanjing University, Nanjing, Jiangsu China; 49 School of Physics, Shandong University, Jinan, Shandong China; 50 Shanghai Key Laboratory for Particle Physics and Cosmology, Department of Physics and Astronomy, Shanghai Jiao Tong University, Shanghai, China; 51 Physics Department, Tsinghua University, Beijing, 100084 China; 52 Laboratoire de Physique Corpusculaire, Clermont Université and Université Blaise Pascal and CNRS/IN2P3, Clermont-Ferrand, France; 53 Nevis Laboratory, Columbia University, Irvington, NY USA; 54 Niels Bohr Institute, University of Copenhagen, Copenhagen, Denmark; 55 INFN Gruppo Collegato di Cosenza, Laboratori Nazionali di Frascati, Frascati, Italy; 56 Dipartimento di Fisica, Università della Calabria, Rende, Italy; 57 Faculty of Physics and Applied Computer Science, AGH University of Science and Technology, Kraków, Poland; 58 Marian Smoluchowski Institute of Physics, Jagiellonian University, Kraków, Poland; 59 Institute of Nuclear Physics, Polish Academy of Sciences, Kraków, Poland; 60 Physics Department, Southern Methodist University, Dallas, TX USA; 61 Physics Department, University of Texas at Dallas, Richardson, TX USA; 62 DESY, Hamburg and Zeuthen, Germany; 63 Institut für Experimentelle Physik IV, Technische Universität Dortmund, Dortmund, Germany; 64 Institut für Kern- und Teilchenphysik, Technische Universität Dresden, Dresden, Germany; 65 Department of Physics, Duke University, Durham, NC USA; 66 SUPA-School of Physics and Astronomy, University of Edinburgh, Edinburgh, UK; 67 INFN Laboratori Nazionali di Frascati, Frascati, Italy; 68 Fakultät für Mathematik und Physik, Albert-Ludwigs-Universität, Freiburg, Germany; 69 Section de Physique, Université de Genève, Geneva, Switzerland; 70 INFN Sezione di Genova, Genoa, Italy; 71 Dipartimento di Fisica, Università di Genova, Genoa, Italy; 72 E. Andronikashvili Institute of Physics, Iv. Javakhishvili Tbilisi State University, Tbilisi, Georgia; 73 High Energy Physics Institute, Tbilisi State University, Tbilisi, Georgia; 74 II Physikalisches Institut, Justus-Liebig-Universität Giessen, Giessen, Germany; 75 SUPA-School of Physics and Astronomy, University of Glasgow, Glasgow, UK; 76 II Physikalisches Institut, Georg-August-Universität, Göttingen, Germany; 77 Laboratoire de Physique Subatomique et de Cosmologie, Université Grenoble-Alpes, CNRS/IN2P3, Grenoble, France; 78 Department of Physics, Hampton University, Hampton, VA USA; 79 Laboratory for Particle Physics and Cosmology, Harvard University, Cambridge, MA USA; 80 Kirchhoff-Institut für Physik, Ruprecht-Karls-Universität Heidelberg, Heidelberg, Germany; 81 Physikalisches Institut, Ruprecht-Karls-Universität Heidelberg, Heidelberg, Germany; 82 ZITI Institut für technische Informatik, Ruprecht-Karls-Universität Heidelberg, Mannheim, Germany; 83 Faculty of Applied Information Science, Hiroshima Institute of Technology, Hiroshima, Japan; 84 Department of Physics, The Chinese University of Hong Kong, Shatin, NT Hong Kong; 85 Department of Physics, The University of Hong Kong, Hong Kong, China; 86 Department of Physics, The Hong Kong University of Science and Technology, Clear Water Bay, Kowloon, Hong Kong China; 87 Department of Physics, Indiana University, Bloomington, IN USA; 88 Institut für Astro- und Teilchenphysik, Leopold-Franzens-Universität, Innsbruck, Austria; 89 University of Iowa, Iowa City, IA USA; 90 Department of Physics and Astronomy, Iowa State University, Ames, IA USA; 91 Joint Institute for Nuclear Research, JINR Dubna, Dubna, Russia; 92 KEK, High Energy Accelerator Research Organization, Tsukuba, Japan; 93 Graduate School of Science, Kobe University, Kobe, Japan; 94 Faculty of Science, Kyoto University, Kyoto, Japan; 95 Kyoto University of Education, Kyoto, Japan; 96 Department of Physics, Kyushu University, Fukuoka, Japan; 97 Instituto de Física La Plata, Universidad Nacional de La Plata and CONICET, La Plata, Argentina; 98 Physics Department, Lancaster University, Lancaster, UK; 99 INFN Sezione di Lecce, Lecce, Italy; 100 Dipartimento di Matematica e Fisica, Università del Salento, Lecce, Italy; 101 Oliver Lodge Laboratory, University of Liverpool, Liverpool, UK; 102 Department of Physics, Jožef Stefan Institute and University of Ljubljana, Ljubljana, Slovenia; 103 School of Physics and Astronomy, Queen Mary University of London, London, UK; 104 Department of Physics, Royal Holloway University of London, Surrey, UK; 105 Department of Physics and Astronomy, University College London, London, UK; 106 Louisiana Tech University, Ruston, LA USA; 107 Laboratoire de Physique Nucléaire et de Hautes Energies, UPMC and Université Paris-Diderot and CNRS/IN2P3, Paris, France; 108 Fysiska institutionen, Lunds universitet, Lund, Sweden; 109 Departamento de Fisica Teorica C-15, Universidad Autonoma de Madrid, Madrid, Spain; 110 Institut für Physik, Universität Mainz, Mainz, Germany; 111 School of Physics and Astronomy, University of Manchester, Manchester, UK; 112 CPPM, Aix-Marseille Université and CNRS/IN2P3, Marseille, France; 113 Department of Physics, University of Massachusetts, Amherst, MA USA; 114 Department of Physics, McGill University, Montreal, QC Canada; 115 School of Physics, University of Melbourne, Melbourne, VIC Australia; 116 Department of Physics, The University of Michigan, Ann Arbor, MI USA; 117 Department of Physics and Astronomy, Michigan State University, East Lansing, MI USA; 118 INFN Sezione di Milano, Milan, Italy; 119 Dipartimento di Fisica, Università di Milano, Milan, Italy; 120 B.I. Stepanov Institute of Physics, National Academy of Sciences of Belarus, Minsk, Republic of Belarus; 121 National Scientific and Educational Centre for Particle and High Energy Physics, Minsk, Republic of Belarus; 122 Department of Physics, Massachusetts Institute of Technology, Cambridge, MA USA; 123 Group of Particle Physics, University of Montreal, Montreal, QC Canada; 124 P.N. Lebedev Physical Institute of the Russian, Academy of Sciences, Moscow, Russia; 125 Institute for Theoretical and Experimental Physics (ITEP), Moscow, Russia; 126 National Research Nuclear University MEPhI, Moscow, Russia; 127 D.V. Skobeltsyn Institute of Nuclear Physics, M.V. Lomonosov Moscow State University, Moscow, Russia; 128 Fakultät für Physik, Ludwig-Maximilians-Universität München, Munich, Germany; 129 Max-Planck-Institut für Physik (Werner-Heisenberg-Institut), Munich, Germany; 130 Nagasaki Institute of Applied Science, Nagasaki, Japan; 131 Graduate School of Science and Kobayashi-Maskawa Institute, Nagoya University, Nagoya, Japan; 132 INFN Sezione di Napoli, Naples, Italy; 133 Dipartimento di Fisica, Università di Napoli, Naples, Italy; 134 Department of Physics and Astronomy, University of New Mexico, Albuquerque, NM USA; 135 Institute for Mathematics, Astrophysics and Particle Physics, Radboud University Nijmegen/Nikhef, Nijmegen, The Netherlands; 136 Nikhef National Institute for Subatomic Physics and University of Amsterdam, Amsterdam, The Netherlands; 137 Department of Physics, Northern Illinois University, DeKalb, IL USA; 138 Budker Institute of Nuclear Physics, SB RAS, Novosibirsk, Russia; 139 Department of Physics, New York University, New York, NY USA; 140 Ohio State University, Columbus, OH USA; 141 Faculty of Science, Okayama University, Okayama, Japan; 142 Homer L. Dodge Department of Physics and Astronomy, University of Oklahoma, Norman, OK USA; 143 Department of Physics, Oklahoma State University, Stillwater, OK USA; 144 Palacký University, RCPTM, Olomouc, Czech Republic; 145 Center for High Energy Physics, University of Oregon, Eugene, OR USA; 146 LAL, Univ. Paris-Sud, CNRS/IN2P3, Université Paris Saclay, Orsay, France; 147 Graduate School of Science, Osaka University, Osaka, Japan; 148 Department of Physics, University of Oslo, Oslo, Norway; 149 Department of Physics, Oxford University, Oxford, UK; 150 INFN Sezione di Pavia, Pavia, Italy; 151 Dipartimento di Fisica, Università di Pavia, Pavia, Italy; 152 Department of Physics, University of Pennsylvania, Philadelphia, PA USA; 153 National Research Centre “Kurchatov Institute” B.P.Konstantinov Petersburg Nuclear Physics Institute, St. Petersburg, Russia; 154 INFN Sezione di Pisa, Pisa, Italy; 155 Dipartimento di Fisica E. Fermi, Università di Pisa, Pisa, Italy; 156 Department of Physics and Astronomy, University of Pittsburgh, Pittsburgh, PA USA; 157 Laboratório de Instrumentação e Física Experimental de Partículas-LIP, Lisboa, Portugal; 158 Faculdade de Ciências, Universidade de Lisboa, Lisboa, Portugal; 159 Department of Physics, University of Coimbra, Coimbra, Portugal; 160 Centro de Física Nuclear da Universidade de Lisboa, Lisbon, Portugal; 161 Departamento de Fisica, Universidade do Minho, Braga, Portugal; 162 Departamento de Fisica Teorica y del Cosmos and CAFPE, Universidad de Granada, Granada, Spain; 163 Dep Fisica and CEFITEC of Faculdade de Ciencias e Tecnologia, Universidade Nova de Lisboa, Caparica, Portugal; 164 Institute of Physics, Academy of Sciences of the Czech Republic, Praha, Czech Republic; 165 Czech Technical University in Prague, Praha, Czech Republic; 166 Faculty of Mathematics and Physics, Charles University in Prague, Praha, Czech Republic; 167 State Research Center Institute for High Energy Physics, (Protvino), NRC KI, Russia; 168 Particle Physics Department, Rutherford Appleton Laboratory, Didcot, UK; 169 INFN Sezione di Roma, Rome, Italy; 170 Dipartimento di Fisica, Sapienza Università di Roma, Rome, Italy; 171 INFN Sezione di Roma Tor Vergata, Rome, Italy; 172 Dipartimento di Fisica, Università di Roma Tor Vergata, Rome, Italy; 173 INFN Sezione di Roma Tre, Rome, Italy; 174 Dipartimento di Matematica e Fisica, Università Roma Tre, Rome, Italy; 175 Faculté des Sciences Ain Chock, Réseau Universitaire de Physique des Hautes Energies-Université Hassan II, Casablanca, Morocco; 176 Centre National de l’Energie des Sciences Techniques Nucleaires, Rabat, Morocco; 177 Faculté des Sciences Semlalia, Université Cadi Ayyad, LPHEA-Marrakech, Marrakech, Morocco; 178 Faculté des Sciences, Université Mohamed Premier and LPTPM, Oujda, Morocco; 179 Faculté des Sciences, Université Mohammed V, Rabat, Morocco; 180 DSM/IRFU (Institut de Recherches sur les Lois Fondamentales de l’Univers), CEA Saclay (Commissariat à l’Energie Atomique et aux Energies Alternatives), Gif-sur-Yvette, France; 181 Santa Cruz Institute for Particle Physics, University of California Santa Cruz, Santa Cruz, CA USA; 182 Department of Physics, University of Washington, Seattle, WA USA; 183 Department of Physics and Astronomy, University of Sheffield, Sheffield, UK; 184 Department of Physics, Shinshu University, Nagano, Japan; 185 Fachbereich Physik, Universität Siegen, Siegen, Germany; 186 Department of Physics, Simon Fraser University, Burnaby, BC Canada; 187 SLAC National Accelerator Laboratory, Stanford, CA USA; 188 Faculty of Mathematics, Physics and Informatics, Comenius University, Bratislava, Slovak Republic; 189 Department of Subnuclear Physics, Institute of Experimental Physics of the Slovak Academy of Sciences, Kosice, Slovak Republic; 190 Department of Physics, University of Cape Town, Cape Town, South Africa; 191 Department of Physics, University of Johannesburg, Johannesburg, South Africa; 192 School of Physics, University of the Witwatersrand, Johannesburg, South Africa; 193 Department of Physics, Stockholm University, Stockholm, Sweden; 194 The Oskar Klein Centre, Stockholm, Sweden; 195 Physics Department, Royal Institute of Technology, Stockholm, Sweden; 196 Departments of Physics and Astronomy and Chemistry, Stony Brook University, Stony Brook, NY USA; 197 Department of Physics and Astronomy, University of Sussex, Brighton, UK; 198 School of Physics, University of Sydney, Sydney, Australia; 199 Institute of Physics, Academia Sinica, Taipei, Taiwan; 200 Department of Physics, Technion: Israel Institute of Technology, Haifa, Israel; 201 Raymond and Beverly Sackler School of Physics and Astronomy, Tel Aviv University, Tel Aviv, Israel; 202 Department of Physics, Aristotle University of Thessaloniki, Thessaloníki, Greece; 203 International Center for Elementary Particle Physics and Department of Physics, The University of Tokyo, Tokyo, Japan; 204 Graduate School of Science and Technology, Tokyo Metropolitan University, Tokyo, Japan; 205 Department of Physics, Tokyo Institute of Technology, Tokyo, Japan; 206 Department of Physics, University of Toronto, Toronto, ON Canada; 207 TRIUMF, Vancouver, BC Canada; 208 Department of Physics and Astronomy, York University, Toronto, ON Canada; 209 Faculty of Pure and Applied Sciences, and Center for Integrated Research in Fundamental Science and Engineering, University of Tsukuba, Tsukuba, Japan; 210 Department of Physics and Astronomy, Tufts University, Medford, MA USA; 211 Centro de Investigaciones, Universidad Antonio Narino, Bogota, Colombia; 212 Department of Physics and Astronomy, University of California Irvine, Irvine, CA USA; 213 INFN Gruppo Collegato di Udine, Sezione di Trieste, Udine, Italy; 214 ICTP, Trieste, Italy; 215 Dipartimento di Chimica Fisica e Ambiente, Università di Udine, Udine, Italy; 216 Department of Physics, University of Illinois, Urbana, IL USA; 217 Department of Physics and Astronomy, University of Uppsala, Uppsala, Sweden; 218 Instituto de Física Corpuscular (IFIC) and Departamento de Física Atómica, Molecular y Nuclear and Departamento de Ingeniería Electrónica and Instituto de Microelectrónica de Barcelona (IMB-CNM), University of Valencia and CSIC, Valencia, Spain; 219 Department of Physics, University of British Columbia, Vancouver, BC Canada; 220 Department of Physics and Astronomy, University of Victoria, Victoria, BC Canada; 221 Department of Physics, University of Warwick, Coventry, UK; 222 Waseda University, Tokyo, Japan; 223 Department of Particle Physics, The Weizmann Institute of Science, Rehovot, Israel; 224 Department of Physics, University of Wisconsin, Madison, WI USA; 225 Fakultät für Physik und Astronomie, Julius-Maximilians-Universität, Würzburg, Germany; 226 Fakultät für Mathematik und Naturwissenschaften, Fachgruppe Physik, Bergische Universität Wuppertal, Wuppertal, Germany; 227 Department of Physics, Yale University, New Haven, CT USA; 228 Yerevan Physics Institute, Yerevan, Armenia; 229 Centre de Calcul de l’Institut National de Physique Nucléaire et de Physique des Particules (IN2P3), Villeurbanne, France; 230 CERN, Geneva, Switzerland

## Abstract

This paper presents a new method of reconstructing the individual charged and neutral hadrons in tau decays with the ATLAS detector. The reconstructed hadrons are used to classify the decay mode and to calculate the visible four-momentum of reconstructed tau candidates, significantly improving the resolution with respect to the calibration in the existing tau reconstruction. The performance of the reconstruction algorithm is optimised and evaluated using simulation and validated using samples of $Z\rightarrow \tau \tau$ and $Z(\rightarrow \mu \mu )$+jets events selected from proton–proton collisions at a centre-of-mass energy $\sqrt{s}=8\,\text {TeV}$, corresponding to an integrated luminosity of 5 $\mathrm{fb}^{-1}$.

## Introduction

Final states with hadronically decaying tau leptons play an important part in the physics programme of the ATLAS experiment [1]. Examples from Run 1 (2009–2013) of the Large Hadron Collider (LHC) [2] are measurements of Standard Model processes [3–7], Higgs boson searches [8], including models with extended Higgs sectors [9–11], and searches for new physics phenomena, such as supersymmetry [12–14], new heavy gauge bosons [15] and leptoquarks [16]. These analyses depended on robust tau reconstruction and excellent particle identification algorithms that provided suppression of backgrounds from jets, electrons and muons [17].

With the discovery of a Higgs boson [18, 19] and evidence for the Higgs-boson Yukawa coupling to tau leptons [8, 20], a key future measurement will be that of the *CP* mixture of the Higgs boson via spin effects in $H\rightarrow \tau \tau$ decays [21–23]. This measurement relies on high-purity selection of the $\tau ^{-} \rightarrow \pi ^{-} \nu$, $\tau ^{-} \rightarrow \pi ^{-} \pi ^0 \nu$ and $\tau ^{-} \rightarrow \pi ^{-} \pi ^+ \pi ^{-} \nu$ decays, as well as the reconstruction of the individual charged and neutral pion four-momenta. The tau reconstruction used in ATLAS throughout Run 1 (here denoted as “Baseline”), however, only differentiates tau decay modes by the number of charged hadrons and does not provide access to reconstructed neutral pions.

This paper presents a new method (called “Tau Particle Flow ”) of reconstructing the individual charged and neutral hadrons in tau decays with the ATLAS detector. Charged hadrons are reconstructed from their tracks in the tracking system. Neutral pions are reconstructed from their energy deposits in the calorimeter. The reconstructed hadrons, which make up the visible part of the tau decay ($\tau _{\mathrm{had}{\text {-}}\mathrm{vis}}$), are used to classify the decay mode and to calculate the four-momentum of reconstructed $\tau _{\mathrm{had}{\text {-}}\mathrm{vis}}$ candidates. The superior four-momentum resolution from the tracking system compared to the calorimeter, for charged hadrons with transverse momentum ($p_{\text {T}}$) less than $\sim$100$\,\text {GeV}$, leads to a significant improvement in the tau energy and directional resolution. This improvement, coupled with the ability to better identify the hadronic tau decay modes, could lead to better resolution of the ditau mass reconstruction [24]. The performance of the Tau Particle Flow is validated using samples of real hadronic tau decays and jets in *Z*+jets events selected from data. The samples correspond to $5\,\mathrm{fb}^{-1}$ of data collected during proton–proton collisions at a centre-of-mass energy of $\sqrt{s} = 8\,\text {TeV}$, which was the amount of data reprocessed using Tau Particle Flow. While similar concepts for the reconstruction of hadronic tau decays have been employed at other experiments [25–31], the Tau Particle Flow is specifically designed to exploit the features of the ATLAS detector and to perform well in the environment of the LHC.

The paper is structured as follows. The ATLAS detector, event samples, and the reconstruction of physics objects used to select $\tau _{\mathrm{had}{\text {-}}\mathrm{vis}}$ candidates from the $8\,\text {TeV}$ data are described in Sect. 2. The properties of $\tau _{\mathrm{had}{\text {-}}\mathrm{vis}}$ decays and the Tau Particle Flow method are described in Sect. 3, including its concepts (Sect. 3.1), neutral pion reconstruction (Sect. 3.2), reconstruction of individual photon energy deposits (Sect. 3.3), decay mode classification (Sect. 3.4) and $\tau _{\mathrm{had}{\text {-}}\mathrm{vis}}$ four-momentum reconstruction (Sect. 3.5). Conclusions are presented in Sect. 4.

## ATLAS detector and event samples

### The ATLAS detector

The ATLAS detector [1] consists of an inner tracking system surrounded by a superconducting solenoid, electromagnetic (EM) and hadronic (HAD) calorimeters, and a muon spectrometer. The inner detector is immersed in a 2 T axial magnetic field, and consists of pixel and silicon microstrip detectors inside a transition radiation tracker, which together provide charged-particle tracking in the region $|\eta |<2.5$.^1^ The EM calorimeter is based on lead and liquid argon as absorber and active material, respectively. In the central rapidity region, the EM calorimeter is divided radially into three layers: the innermost layer (EM1) is finely segmented in $\eta$ for optimal $\gamma / \pi ^0$ separation, the layer next in radius (EM2) collects most of the energy deposited by electron and photon showers, and the third layer (EM3) is used to correct leakage beyond the EM calorimeter for high-energy showers. A thin presampler layer (PS) in front of EM1 and in the range $|\eta | < 1.8$ is used to correct showers for upstream energy loss. Hadron calorimetry is based on different detector technologies, with scintillator tiles ($|\eta | < 1.7$) or liquid argon ($1.5 < |\eta | < 4.9$) as active media, and with steel, copper, or tungsten as absorber material. The calorimeters provide coverage within $|\eta |<4.9$. The muon spectrometer consists of superconducting air-core toroids, a system of trigger chambers covering the range $|\eta |<2.4$, and high-precision tracking chambers allowing muon momentum measurements within $|\eta |<2.7$. A three-level trigger system is used to select interesting events [32]. The first-level trigger is implemented in hardware and uses a subset of detector information to reduce the event rate to a design value of at most 75 kHz. This is followed by two software-based trigger levels which together reduce the average event rate to 400 Hz.

### Physics objects

This section describes the Baseline $\tau _{\mathrm{had}{\text {-}}\mathrm{vis}}$ reconstruction and also the reconstruction of muons and the missing transverse momentum, which are required for the selection of samples from data. Tau Particle Flow operates on each reconstructed Baseline tau candidate to reconstruct the charged and neutral hadrons, classify the decay mode and to provide an alternative $\tau _{\mathrm{had}{\text {-}}\mathrm{vis}}$ four-momentum. Suppression of backgrounds from other particles misidentified as $\tau _{\mathrm{had}{\text {-}}\mathrm{vis}}$ is achieved independently of the Tau Particle Flow.

The Baseline $\tau _{\mathrm{had}{\text {-}}\mathrm{vis}}$ reconstruction and energy calibration, and the algorithms used to suppress backgrounds from jets, electrons and muons are described in detail in Ref. [17]. Candidates for hadronic tau decays are built from jets reconstructed using the anti-$k_{t}$ algorithm [33, 34] with a radius parameter value of 0.4. Three-dimensional clusters of calorimeter cells calibrated using a local hadronic calibration [35, 36] serve as inputs to the jet algorithm. The calculation of the $\tau _{\mathrm{had}{\text {-}}\mathrm{vis}}$ four-momentum uses clusters within the *core region* ($\Delta R<0.2$ from the initial jet-axis). It includes a final tau-specific calibration derived from simulated samples, which accounts for out-of-cone energy, underlying event, the typical composition of hadrons in hadronic tau decays and contributions from multiple interactions occurring in the same and neighbouring bunch crossings (called pile-up). Tracks reconstructed in the inner detector are matched to the $\tau _{\mathrm{had}{\text {-}}\mathrm{vis}}$ candidate if they are in the core region and satisfy the following criteria: $p_{\text {T}} >1\,\text {GeV}$, at least two associated hits in the pixel layers of the inner detector, and at least seven hits in total in the pixel and silicon microstrip layers. Furthermore, requirements are imposed on the distance of closest approach of the tracks to the tau primary vertex in the transverse plane, $|d_0|<1.0$ mm, and longitudinally, $|z_0\sin \theta |<1.5$ mm. The $\tau _{\mathrm{had}{\text {-}}\mathrm{vis}}$ charge is reconstructed from the sum of the charges of the associated tracks.

Backgrounds for $\tau _{\mathrm{had}{\text {-}}\mathrm{vis}}$ candidates originating from quark- and gluon-initiated jets are discriminated against by combining shower shape and tracking information in a multivariate algorithm that employs boosted decision trees (BDTs) [37]. The efficiency of the jet discrimination algorithm has little dependence on the $p_{\text {T}}$ of the $\tau _{\mathrm{had}{\text {-}}\mathrm{vis}}$ candidates (evaluated using candidates with $p_{\text {T}} >15\,\text {GeV}$) or on the number of reconstructed primary vertices, which is correlated to the amount of pile-up, and has been evaluated up to a maximum of 25 primary vertices per event. All $\tau _{\mathrm{had}{\text {-}}\mathrm{vis}}$ candidates are required to have $p_{\text {T}} {} > 15\,\text {GeV}$, to be in the fiducial volume of the inner detector, $|\eta | < 2.5$, and to have one or three associated tracks. They must also meet jet discrimination criteria, corresponding to an efficiency of about 55 % (40 %) for simulated $\tau _{\mathrm{had}{\text {-}}\mathrm{vis}}$ with one (three) charged decay products [17], leading to a rate of false identification for quark- and gluon-initiated jets of below a percent. A discriminant designed to suppress candidates arising from the misidentification of electrons [17] is also applied.

Muons are reconstructed using tracks in the muon spectrometer and inner detector [38]. The missing transverse momentum is computed from the combination of all reconstructed and fully calibrated physics objects and the remaining clustered energy deposits in the calorimeter not associated with those objects [39].

### Event samples and selection

The optimisation and measurement of the $\tau _{\mathrm{had}{\text {-}}\mathrm{vis}}$ reconstruction performance requires Monte Carlo simulated events. Samples of simulated *pp* collision events at $\sqrt{s}=8\,\text {TeV}$ are summarised in Table 1. Tau decays are provided by $Z \rightarrow \tau \tau$ events. The *sophisticated tau decay* option of Pythia 8 is used, which provides fully modelled hadronic decays with spin correlations [40]. Tau decays in the $t\bar{t}$ sample are generated by Tauola [41]. Photon radiation is performed by Photos [42]. Single-pion samples are also used, in which the pions originate from the centre of the ATLAS detector and are generated to have a uniform distribution in $\phi$ and $\eta$ ($|\eta |<5.5$) and also in $\log (E)$ ($200\,\text {MeV}< E < 2\,\text {TeV}$).

**Table 1:** Details regarding the simulated samples of *pp* collision events. The following information is provided for each sample: the generator of the hard interaction, parton shower, hadronisation and multiple parton interactions; the set of parton distribution functions (PDFs) and the underlying event (UE) tune of the Monte Carlo

Process	Generator	PDFs	UE tune
$Z \rightarrow \tau \tau$	Pythia 8 [43]	CTEQ6L1 [44]	AU2 [45]
$W\rightarrow \mu \nu$	Alpgen [46]+Pythia 8	CTEQ6L1	Perugia [47]
$W \rightarrow \tau \nu$	Alpgen+Pythia 8	CTEQ6L1	Perugia
$Z \rightarrow \mu \mu$	Alpgen+Pythia 8	CTEQ6L1	Perugia
$t\bar{t}$	MC@NLO [48–50]+Herwig [51, 52]	CT10 [53]	AUET2 [45]

The response of the ATLAS detector is simulated using Geant4 [54, 55] with the hadronic-shower model QGSP_BERT [56, 57]. The parameters of the underlying event (UE) simulation were tuned using collision data. Simulated *pp* collision events are overlaid with additional minimum-bias events generated with Pythia 8 to account for the effect of pile-up. When comparing to the data, the simulated events are reweighted so that the distribution of the number of pile-up interactions matches that in the data. The simulated events are reconstructed with the same algorithm chain as used for the collision data.

Samples of $\tau _{\mathrm{had}{\text {-}}\mathrm{vis}}$ candidates are selected from the data using a *tag-and-probe* approach. Candidates originating from hadronic tau decays and jets are obtained by selecting $Z \rightarrow \tau \tau$ and $Z(\rightarrow \mu \mu )\mathrm{+jets}$ events, respectively. The data were collected by the ATLAS detector during *pp* collisions at $\sqrt{s}=8\,\text {TeV}$. The sample corresponds to an integrated luminosity of 5 fb$^{-1}$ after making suitable data quality requirements for the operation of the tracking, calorimeter, and muon spectrometer subsystems. The data have a maximum instantaneous luminosity of $7\cdot 10^{33}$ cm$^{-2}$ s$^{-1}$ and an average number of 19 *pp* interactions in the same bunch crossing.

The $Z \rightarrow \tau \tau$ tag-and-probe approach follows Ref. [17]; events are triggered by the presence of a muon from a leptonic tau decay (*tag*) and must contain a $\tau _{\mathrm{had}{\text {-}}\mathrm{vis}}$ candidate (*probe*) with $p_{\text {T}} {} > 20\,\text {GeV}$, which is used to evaluate the tau reconstruction performance. The $\tau _{\mathrm{had}{\text {-}}\mathrm{vis}}$ selection criteria described in Sect. 2.2 are used. In addition the $\tau _{\mathrm{had}{\text {-}}\mathrm{vis}}$ must have unit charge which is opposite to that of the muon. A discriminant designed to suppress candidates arising from the misidentification of muons [17] is also applied to increase signal purity. The invariant mass of the muon and $\tau _{\mathrm{had}{\text {-}}\mathrm{vis}}$, $m(\mu ,\tau _{\mathrm{had}{\text {-}}\mathrm{vis}})$, is required to be in the range $50\,\text {GeV}< m(\mu ,\tau _{\mathrm{had}{\text {-}}\mathrm{vis}}) < 85\,\text {GeV}$, as expected for $Z \rightarrow \tau \tau$ decays. The background is dominated by multijet and $W(\rightarrow \mu \nu )\mathrm{+jets}$ production and is estimated using the techniques from Ref. [7].

The $Z(\rightarrow \mu \mu )\mathrm{+jets}$ tag-and-probe approach follows Ref. [58], with the following differences: both muons are required to have $p_{\text {T}} >26$ $\,\text {GeV}$, the dimuon invariant mass must be between 81 and 101 $\,\text {GeV}$, and the highest-$p_{\text {T}}$ jet is selected as a probe $\tau _{\mathrm{had}{\text {-}}\mathrm{vis}}$ candidate if it satisfies the $\tau _{\mathrm{had}{\text {-}}\mathrm{vis}}$ selection criteria described in Sect. 2.2 but with $p_{\text {T}} {} > 20\,\text {GeV}$ and without the electron discriminant. In this approach, two more steps are made when comparing simulated events to the data. Before the $\tau _{\mathrm{had}{\text {-}}\mathrm{vis}}$ selection, the simulated events are reweighted so that the $p_{\text {T}}$ distribution of the *Z* boson matches that in data. After the full event selection, the overall normalisation of the simulation is scaled to that in the data.

## Reconstruction of the $\tau _{\mathrm{had}{\text {-}}\mathrm{vis}}$

Over 90 % of hadronic tau decays occur through just five dominant decay modes, which yield one or three charged hadrons ($h^{\pm }$), up to two neutral pions ($\pi ^0$) and a tau neutrino. The neutrino goes undetected and is omitted in further discussion of the decay modes. Table 2 gives the following details for each of the five decay modes: the branching fraction, $\mathcal {B}$; the fraction of simulated $\tau _{\mathrm{had}{\text {-}}\mathrm{vis}}$ candidates that pass the $\tau _{\mathrm{had}{\text {-}}\mathrm{vis}}$ selection described in Sect. 2.2 without the jet and electron discrimination, $\mathcal {A}\cdot \varepsilon _\mathrm{reco}$; and the fraction of those that also pass the jet and electron discrimination, $\varepsilon _\mathrm{ID}$. The $h^{\pm }$ ’s are predominantly $\pi ^{\pm }$’s with a minor contribution from $K^{\pm }$’s. The modes with two or three pions proceed mainly through the intermediate $\rho$ or $a_1$ resonances, respectively. The $h^{\pm }$ ’s are sufficiently long-lived that they typically interact with the detector before decaying and are therefore considered stable in the Tau Particle Flow. The $\pi ^0$’s decay almost exclusively to a pair of photons. Approximately half of the photons convert into an $e^+e^-$ pair because of interactions with the beampipe or inner-detector material. Modes with more $\pi ^0$’s tend to have lower $\varepsilon _\mathrm{ID}$ as they have wider showers that are more similar to those produced by quark- and gluon-initiated jets. The mode dependence of $\mathcal {A}\cdot \varepsilon _\mathrm{reco}$ is due to a mixture of effects. The fraction of energy carried by visible decay products is mode dependent and the response of the calorimeter to $h^{\pm }$ ’s and $\pi ^0$’s is different, both of which impact the efficiency of the $\tau _{\mathrm{had}{\text {-}}\mathrm{vis}}$ $p_{\text {T}}$ requirement. The efficiency of the track association is also dependent on the number of $h^{\pm }$ ’s and to a lesser extent the number of $\pi ^0$’s, which can contribute tracks from conversion electrons.

The goal of the Tau Particle Flow is to classify the five decay modes and to reconstruct the individual $h^{\pm }$ ’s and $\pi ^0$’s. The performance is evaluated using the energy and directional residuals of $\pi ^0$ and $\tau _{\mathrm{had}{\text {-}}\mathrm{vis}}$ and the efficiency of the $\tau _{\mathrm{had}{\text {-}}\mathrm{vis}}$ decay mode classification. The $\eta$ and $\phi$ residuals are defined with respect to the generated values: $\eta - \eta ^\mathrm{gen}$ and $\phi - \phi ^\mathrm{gen}$, respectively. For $E_{\text {T}}$, the *relative* residual is defined with respect to the generated value $E_{\text {T}}/E_{\text {T}} ^\mathrm{gen}$. The *core* and *tail* resolutions for $\eta$, $\phi$ and $E_{\text {T}}$ are defined as half of the 68 and 95 % central intervals of their residuals, respectively. Decays into higher-multiplicity states are accommodated by including modes with more than two $\pi ^0$’s in the $h^{\pm } \,{\ge }2\pi ^0$ category and more than one $\pi ^0$ in the $3h^{\pm } \,{\ge }1\pi ^0$ category. Decays with more than three charged hadrons are not considered. No attempt is made to reconstruct neutral kaons or to separate charged kaons from charged pions.

**Table 2:** Five dominant $\tau _{\mathrm{had}{\text {-}}\mathrm{vis}}$ decay modes [59]. Tau neutrinos are omitted from the table. The symbol $h^\pm$ stands for $\pi ^{\pm }$ or $K^\pm$. Decays involving $K^\pm$ contribute $\sim$3 % to the total hadronic branching fraction. Decays involving neutral kaons are excluded. The branching fraction ($\mathcal {B}$), the fraction of generated $\tau _{\mathrm{had}{\text {-}}\mathrm{vis}}$’s in simulated $Z \rightarrow \tau \tau$ events that are reconstructed and pass the $\tau _{\mathrm{had}{\text {-}}\mathrm{vis}}$ selection described in Sect. 2.2 without the jet and electron discrimination ($\mathcal {A}\cdot \varepsilon _\mathrm{reco}$) and the fraction of those $\tau _{\mathrm{had}{\text {-}}\mathrm{vis}}$ candidates that also pass the jet and electron discrimination ($\varepsilon _\mathrm{ID}$) for each decay mode are given

Decay mode	$\mathcal {B}$ (%)	$\mathcal {A}\cdot \varepsilon _\mathrm{reco}$ (%)	$\varepsilon _\mathrm{ID}$ (%)
${h^{\pm }}$	11.5	32	75
${h^{\pm } \,\pi ^0}$	30.0	33	55
${h^{\pm } \,{\ge }2\pi ^0}$	10.6	43	40
${3h^{\pm }}$	9.5	38	70
${3h^{\pm } \,{\ge }1\pi ^0}$	5.1	38	46

### Concepts of the Tau Particle Flow method

The main focus of the Tau Particle Flow method is to reconstruct $\tau _{\mathrm{had}{\text {-}}\mathrm{vis}}$’s with $p_{\text {T}}$ values between 15 and $100\,\text {GeV}$, which is the relevant range for tau leptons produced in decays of electroweak and SM Higgs bosons. In this case the hadrons typically have $p_{\text {T}}$ lower than $20\,\text {GeV}$ (peaked at $\sim$4$\,\text {GeV}$) and have an average separation of $\Delta R\approx 0.07$. The $h^{\pm }$ ’s are reconstructed using the tracking system, from which the charge and momentum are determined. Each track associated with the $\tau _{\mathrm{had}{\text {-}}\mathrm{vis}}$ candidate in the core region is considered to be a $h^{\pm }$ and the $\pi ^{\pm }$ mass hypothesis is applied. Approximately 2 % of the selected $\tau _{\mathrm{had}{\text {-}}\mathrm{vis}}$’s have a misclassified number of $h^{\pm }$ ’s. Overestimation of the number of $h^{\pm }$ ’s is primarily due to additional tracks from conversion electrons, which are highly suppressed by the strict track selection criteria described in Sect. 2.2. Underestimation of the number of $h^{\pm }$ ’s is primarily caused by tracking inefficiencies ($\sim$10 % for charged pions with $p_{\text {T}} >1\,\text {GeV}$ [1]), which arise from interactions of the $h^{\pm }$ ’s with the beampipe or detector material. The $h^{\pm }$ ’s also produce a shower in the calorimeter from which their energy and direction can be determined, but the tracker has a better performance in the relevant momentum range. The shower shapes of $h^{\pm }$ ’s are also highly irregular, with a typical width of $0.02<\Delta R<0.07$ in the EM calorimeter, combined with large fluctuations in the fractional energy depositions in the layers of the calorimeter. The $\pi ^0$’s are reconstructed from their energy deposits in the EM calorimeter. The main challenge is to disentangle their energy deposits from $h^{\pm }$ showers, which have a width similar to the average separation between hadrons. The photons from $\pi ^0$ decays are highly collimated, with a typical separation of $0.01<\Delta R < 0.03$. The majority of the $\pi ^0$ energy is reconstructed in a single cluster in the EM calorimeter. Compared to $h^{\pm }$ ’s, $\pi ^0$ showers are smaller and more regular, leaving on average 10, 30 and 60 % of their energy in PS, EM1 and EM2, respectively. Almost no $\pi ^0$ energy is deposited beyond EM2, so EM3 is considered part of the HAD calorimeter in Tau Particle Flow. The characteristic shower shapes and the kinematics of $h^{\pm }$ ’s and $\pi ^0$’s are used to identify $\pi ^0$’s and to classify the tau decay mode.

In the following sections, the individual steps of the Tau Particle Flow method for $\tau _{\mathrm{had}{\text {-}}\mathrm{vis}}$ reconstruction are described. The first step is the reconstruction and identification of neutral pions. Next, energy deposits from individual photons in the finely segmented EM1 layer are reconstructed to identify cases where two $\pi ^0$’s are contained within a single cluster. The decay mode is then classified by exploiting the available information from the reconstructed $h^{\pm }$ ’s and $\pi ^0$’s and the photons reconstructed in EM1. Following the decay mode classification, the $\tau _{\mathrm{had}{\text {-}}\mathrm{vis}}$ four-momentum is reconstructed from the individual hadrons and then combined with the Baseline energy calibration to reduce tails in the $E_{\text {T}}$ residual distribution. The performance of the Tau Particle Flow is evaluated using $\tau _{\mathrm{had}{\text {-}}\mathrm{vis}}$ candidates from simulated $Z \rightarrow \tau \tau$ events.

### Reconstruction and identification of neutral pions

The reconstruction of neutral pion candidates ($\pi ^0 _\mathrm{cand}$) within hadronic tau decays using the Tau Particle Flow proceeds as follows. First, $\pi ^0 _\mathrm{cand}$’s are created by clustering cells in the EM calorimeter in the core region of the $\tau _{\mathrm{had}{\text {-}}\mathrm{vis}}$. In the next step, the $\pi ^0 _\mathrm{cand}$ energy is corrected for contamination from $h^{\pm }$ ’s. To do this, the energy that each $h^{\pm }$ deposits in the EM calorimeter ($E_{h^{\pm }}^\mathrm{EM}$) is estimated as the difference between the energy of the $h^{\pm }$ from the tracking system ($E_{h^{\pm }}^\mathrm{trk}$) and the energy deposited in the HAD calorimeter which is associated with the $h^{\pm }$ ($E_{h^{\pm }}^\mathrm{HAD}$): $E_{h^{\pm }}^\mathrm{EM}= E_{h^{\pm }}^\mathrm{trk}- E_{h^{\pm }}^\mathrm{HAD}$. To calculate $E_{h^{\pm }}^\mathrm{HAD}$, all clustered energy deposits in the HAD calorimeter in the core region are assigned to the closest $h^{\pm }$, determined using the track position extrapolated to the calorimeter layer that contains most of the cluster energy. The $E_{h^{\pm }}^\mathrm{EM}$ of each $h^{\pm }$ is then subtracted from the energy of the closest $\pi ^0 _\mathrm{cand}$ if it is within $\Delta R =0.04$ of the $h^{\pm }$.

**Fig. 1:**
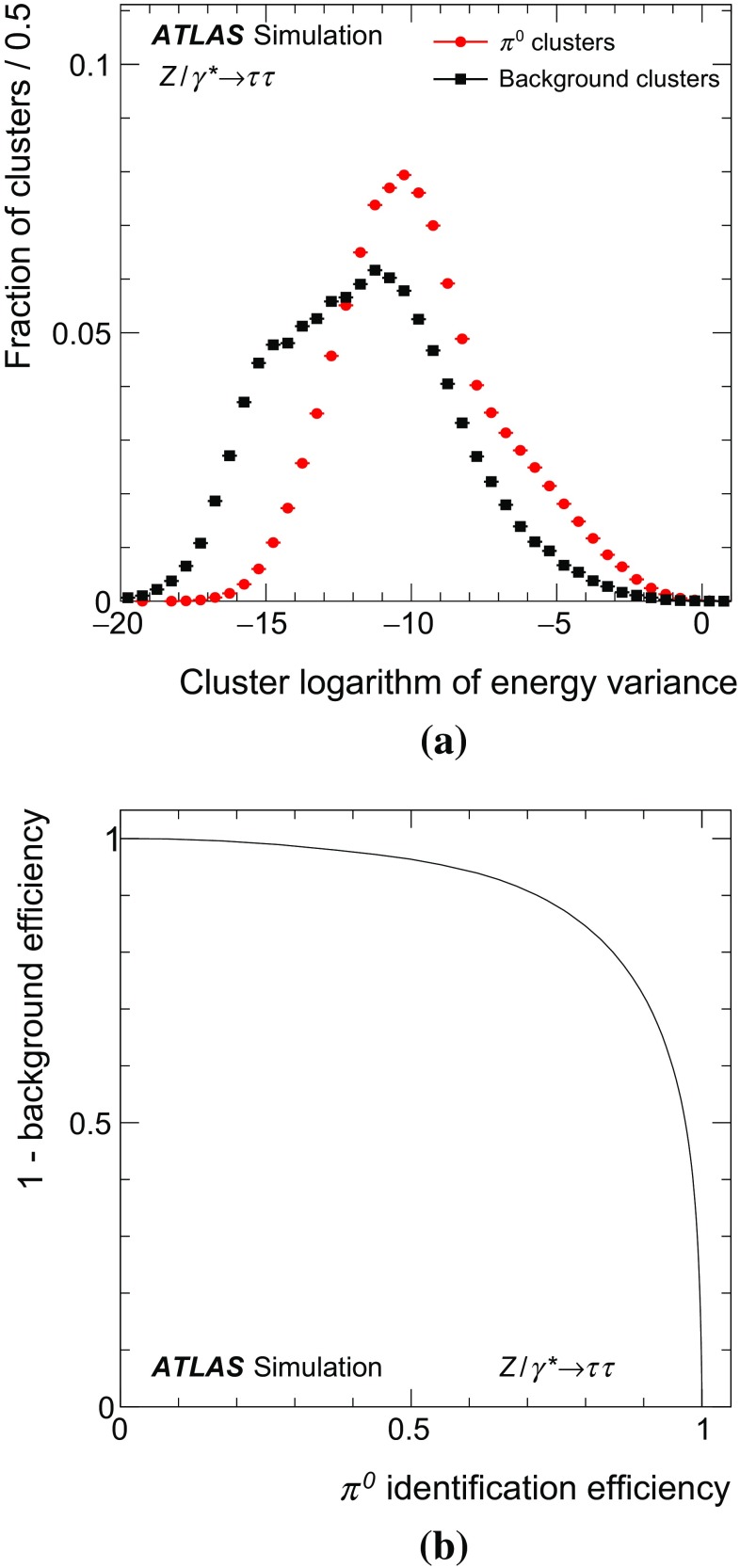
**a** Distribution of the logarithm of the second moment in energy density of $\pi ^0 _\mathrm{cand}$ clusters that do (signal) or do not (background) originate from $\pi ^0$’s, as used in the $\pi ^0$ identification. **b** 1 $-$ efficiency for background $\pi ^0 _\mathrm{cand}$’s vs. the efficiency for signal $\pi ^0 _\mathrm{cand}$’s to pass thresholds on the $\pi ^0$ identification score. The $\pi ^0 _\mathrm{cand}$’s in both figures are associated with $\tau _{\mathrm{had}{\text {-}}\mathrm{vis}}$’s selected from simulated $Z \rightarrow \tau \tau$ events

At this stage, many of the $\pi ^0 _\mathrm{cand}$’s in reconstructed hadronic tau decays do not actually originate from $\pi ^0$’s, but rather from $h^{\pm }$ remnants, pile-up or other sources. The purity of $\pi ^0 _\mathrm{cand}$’s is improved by applying a minimum $p_{\text {T}}$ requirement and an identification criterion designed to reject $\pi ^0 _\mathrm{cand}$’s not from $\pi ^0$’s. The $p_{\text {T}}$ thresholds are in the range 2.1–$2.7\,\text {GeV}$. After the $p_{\text {T}}$ requirement the background is dominated by $h^{\pm }$ remnants. The $\pi ^0$ identification uses a BDT and exploits the properties of the $\pi ^0 _\mathrm{cand}$ clusters, such as the energy density and the width and depth of the shower. The variables used for $\pi ^0 _\mathrm{cand}$ identification are described in Table 3. The BDT is trained using $\tau _{\mathrm{had}{\text {-}}\mathrm{vis}}$’s that have only one $h^{\pm }$, and which are produced in simulated $Z \rightarrow \tau \tau$ events. The $\pi ^0 _\mathrm{cand}$’s are assigned to signal or background based on whether or not they originated from a generated $\pi ^0$. Figure 1a shows signal and background distributions for the logarithm of the second moment in energy density, which is one of the more important identification variables. The discriminating power of the $\pi ^0$ identification is quantified by comparing the efficiency of signal and background $\pi ^0 _\mathrm{cand}$’s to pass thresholds on the identification score, as shown in Fig. 1b. The $p_{\text {T}}$ and identification score thresholds are optimised in five $|\eta |$ ranges, corresponding to structurally different regions of the calorimeter, to maximise the number of $\tau _{\mathrm{had}{\text {-}}\mathrm{vis}}$’s with the correct number of reconstructed $h^{\pm }$ ’s and identified $\pi ^0 _\mathrm{cand}$’s  ($\pi ^0 _\mathrm{ID}$’s).

**Fig. 2:**
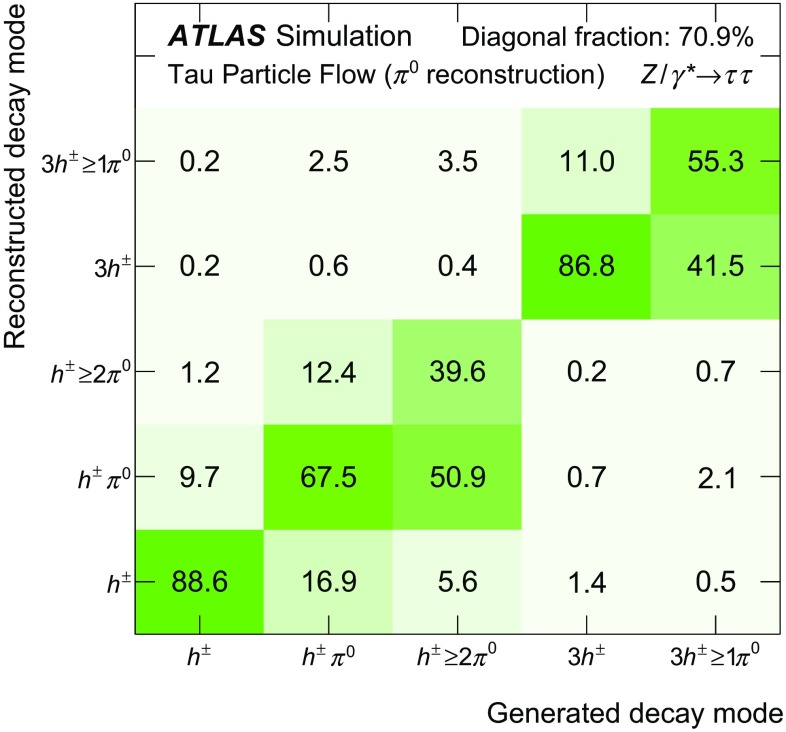
Decay mode classification efficiency matrix showing the probability for a given generated mode to be reconstructed as a particular mode by the Tau Particle Flow after $\pi ^0$ reconstruction in simulated $Z \rightarrow \tau \tau$ events. Decays containing neutral kaons are omitted. Only decays from $\tau _{\mathrm{had}{\text {-}}\mathrm{vis}}$’s that are reconstructed and pass the selection described in Sect. 2.2 are considered. The statistical uncertainty is negligible

The $h^{\pm }$ and $\pi ^0$ counting performance is depicted in Fig. 2 by a *decay mode classification matrix* which shows the probability for a given generated mode to be reconstructed as a particular mode. Only $\tau _{\mathrm{had}{\text {-}}\mathrm{vis}}$ decays that are reconstructed and pass the selection described in Sect. 2.2 are considered (corresponding efficiencies are given in Table 2). The total fraction of correctly classified tau decays (diagonal fraction) is 70.9 %. As can be seen, for $\tau _{\mathrm{had}{\text {-}}\mathrm{vis}}$’s with one $h^{\pm }$, the separation of modes with and without $\pi ^0$’s is quite good, but it is difficult to distinguish between $h^{\pm } \,\pi ^0$ and $h^{\pm } \,{\ge }2\pi ^0$. The largest contributions to the misclassification arise from $h^{\pm } \,{\ge }2\pi ^0$ decays where one of the $\pi ^0$’s failed selection or where the energy deposits of both $\pi ^0$’s merge into a single cluster. It is also difficult to distinguish between the $3h^{\pm }$ and $3h^{\pm } \,{\ge }1\pi ^0$ modes because the $\pi ^0$’s are typically soft with large overlapping $h^{\pm }$ deposits.

**Table 3:** Cluster variables used for $\pi ^0 _\mathrm{cand}$ identification. The variables $|\eta ^\mathrm{clus}|$, $\langle r^2\rangle ^\mathrm{clus}$, $\lambda _\mathrm{centre}^\mathrm{clus}$, $f_\mathrm{core}^\mathrm{clus}$ and $\log \langle \rho ^2\rangle ^\mathrm{clus}$ are taken directly from the cluster reconstruction [36]. To avoid confusion with other variables used in tau reconstruction, the superscript *clus* has been added to each variable

Cluster pseudorapidity, $$|\eta ^\mathrm{clus}|$$
Magnitude of the energy-weighted $\eta$ position of the cluster
Cluster width, $$\langle r^2\rangle ^\mathrm{clus}$$
Second moment in distance to the shower axis
Cluster $\eta$ width in EM1, $\langle \eta ^{2}_\text {EM1}\rangle ^\mathrm{clus}$
Second moment in $\eta$ in EM1
Cluster $\eta$ width in EM2, $\langle \eta ^{2}_\text {EM2}\rangle ^\mathrm{clus}$
Second moment in $\eta$ in EM2
Cluster depth, $$\lambda _\mathrm{centre}^\mathrm{clus}$$
Distance of the shower centre from the calorimeter front face measured along the shower axis
Cluster PS energy fraction, $f_\mathrm{PS}^\mathrm{clus}$
Fraction of energy in the PS
Cluster core energy fraction, $f_\mathrm{core}^\mathrm{clus}$
Sum of the highest cell energy in PS, EM1 and EM2 divided by the total energy
Cluster logarithm of energy variance, $\log \langle \rho ^2\rangle ^\mathrm{clus}$
Logarithm of the second moment in energy density
Cluster EM1 core energy fraction, $f_\mathrm{core,EM1}^\mathrm{clus}$
Energy in the three innermost EM1 cells divided by the total energy in EM1
Cluster asymmetry with respect to track, $\mathcal {A}_\mathrm{track}^\mathrm{clus}$
Asymmetry in $\eta$–$\phi$ space of the energy distribution in EM1 with respect to the extrapolated track position
Cluster EM1 cells, $N_\mathrm{EM1}^\mathrm{clus}$
Number of cells in EM1 with positive energy
Cluster EM2 cells, $N_\mathrm{EM2}^\mathrm{clus}$
Number of cells in EM2 with positive energy

Two alternative methods for $\pi ^0$ reconstruction were also developed. In the first method (Pi0Finder) the number of $\pi ^0$’s in the core region is first estimated from global tau features measured using calorimetric quantities and the momenta of the associated $h^{\pm }$ tracks. Clusters in the EM calorimeter are then chosen as $\pi ^0 _\mathrm{cand}$’s using a $\pi ^0$ likeness score based on their energy deposition in the calorimeter layers and the $\tau _{\mathrm{had}{\text {-}}\mathrm{vis}}$ track momenta. The likeness score does not exploit cluster moments to the same extent as the $\pi ^0$ identification of the Tau Particle Flow and cluster moments are not used at all to estimate the number of $\pi ^0$. This method was used to calculate variables for jet discrimination in Run 1 [17], but was not exploited further. The other method (*shower shape subtraction*, SSS) is a modified version of Tau Particle Flow, which attempts to subtract the $h^{\pm }$ shower from the calorimeter at cell level using average shower shapes derived from simulation. The shower shapes are normalised such that their integral corresponds to $E_{h^{\pm }}^\mathrm{EM}$ and centred on the extrapolated position of the $h^{\pm }$ track. They are then subtracted from the EM calorimeter prior to the clustering, replacing the cluster-level subtraction of $E_{h^{\pm }}^\mathrm{EM}$.

**Fig. 3:**
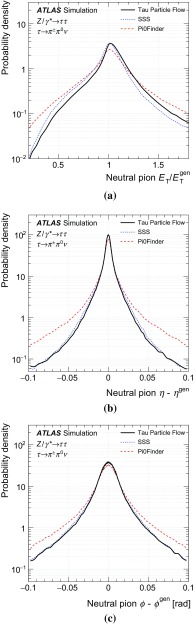
Distributions of the $\pi ^0$ residuals in **a** transverse energy $E_{\text {T}}$, **b** pseudorapidity $\eta$ and **c** azimuth $\phi$ in correctly reconstructed $h^{\pm } \,\pi ^0$ decays of tau leptons in simulated $Z \rightarrow \tau \tau$ events

The $\pi ^0$ $E_{\text {T}}$, $\eta$ and $\phi$ residual distributions for all $\pi ^0$ reconstruction algorithms are shown in Fig. 3a–c, respectively. The core angular resolutions for each algorithm are quite similar with $\sim$0.0056 in $\eta$ and $\sim$0.012 rad in $\phi$. The Pi0Finder algorithm has the poorest performance, with core resolutions of 0.0086 and 0.016 rad in $\eta$ and $\phi$, respectively, and significantly larger tails. The core $E_{\text {T}}$ resolutions are almost identical for the Tau Particle Flow and SSS, both with 16 %, compared to 23 % for Pi0Finder. The Tau Particle Flow and SSS both show a shift in the reconstructed $E_{\text {T}}$ of a few percent, due to incomplete subtraction of the $h^{\pm }$ remnant. In the calculation of the $\tau _{\mathrm{had}{\text {-}}\mathrm{vis}}$ four-momentum in the Tau Particle Flow (Sect. 3.5), this bias is corrected for by a decay-mode-dependent calibration. Despite the more sophisticated shower subtraction employed in the SSS algorithm, it does not perform significantly better; the improvement in the total fraction of correctly classified tau decays is $\sim$1 %. This is partly because many of the $\pi ^0 _\mathrm{cand}$’s are sufficiently displaced from $h^{\pm }$ ’s so that they have little energy contamination and are unaffected by the subtraction, and partly because the signature of clusters that contain $\pi ^0$’s, even in the presence of overlapping $h^{\pm }$ energy, is distinct enough for the BDT to identify. Contributions from pile-up have little effect on the $\pi ^0 _\mathrm{cand}$ reconstruction in Tau Particle Flow; on average the $E_{\text {T}}$ increases by $\sim$15$\,\text {MeV}$ and its resolution degrades fractionally by $\sim$0.5$\,\%$ per additional reconstructed vertex.

### Reconstruction of individual photon energy deposits in EM1

During the $\pi ^0$ reconstruction, the energy deposits from both photons typically merge into a single cluster. Furthermore, for $Z \rightarrow \tau \tau$ events, in about half of the $h^{\pm } \,{\ge }2\pi ^0$ decays misclassified as $h^{\pm } \,\pi ^0$ by the $\pi ^0$ reconstruction, at least three of the photons from two $\pi ^0$’s are grouped into a single cluster. The fraction increases for higher $\tau _{\mathrm{had}{\text {-}}\mathrm{vis}}$ $p_{\text {T}}$ due to the collimation of the tau decay products. The identification of the energy deposits from individual photons in the finely segmented EM1 layer can be exploited to improve the $\pi ^0$ reconstruction, as discussed in the following.

Almost all photons begin to shower by the time they traverse EM1, where they deposit on average $\sim$30 % of their energy. In contrast, particles that do not interact electromagnetically rarely deposit a significant amount of energy in this layer, making it ideal for the identification of photons. Furthermore, the cell segmentation in $\eta$ in this layer is finer than the average photon separation and comparable to the average photon shower width, allowing individual photons to be distinguished.

**Fig. 4:**
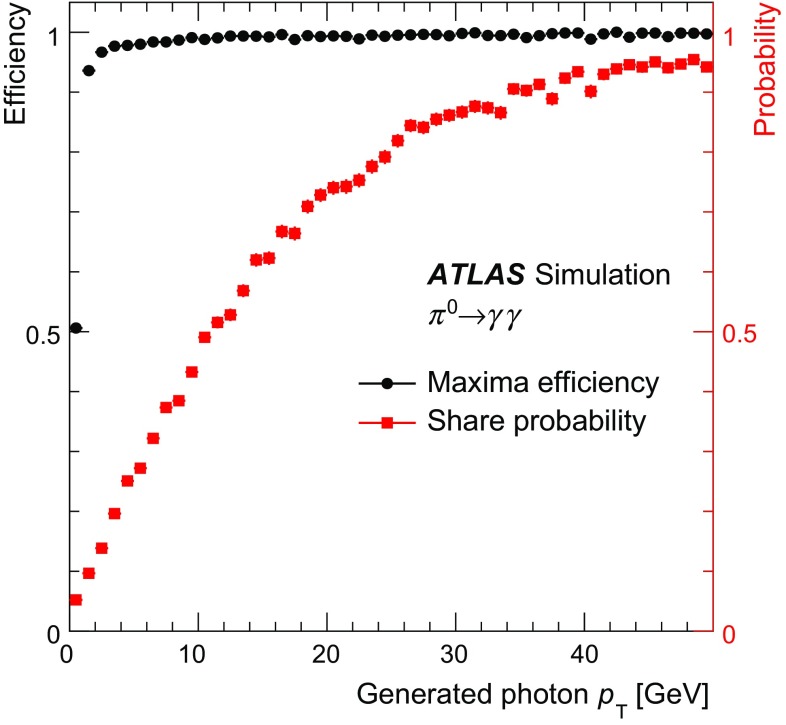
Efficiency for a photon to create a maximum in the first layer of the EM calorimeter in simulated $\pi ^{0}\rightarrow \gamma \gamma$ events and the corresponding probability to create a maximum that is shared with the other photon. The photons are required to not interact with the material in the tracking system

The reconstruction of energy deposits in EM1 proceeds as follows. First, local energy maxima are searched for within the core region. A local maximum is defined as a single cell with $E_{\text {T}} >100\,\text {MeV}$ whose nearest neighbours in $\eta$ both have lower $E_{\text {T}}$. Maxima found in adjacent $\phi$ cells are then combined: their energy is summed and the energy-weighted mean of their $\phi$ positions is used. Figure 4 shows the efficiency for photons to create a local maximum (maxima efficiency), evaluated in the sample of single $\pi ^0$’s. The efficiency decreases rapidly at low photon $p_{\text {T}}$ as many of the photons fall below the $100\,\text {MeV}$ threshold. The fraction of misreconstructed maxima due to noise or fluctuations from the photon shower is very low for maxima with $E_{\text {T}} >500\,\text {MeV}$, but increases quickly at lower $E_{\text {T}}$. At high photon $p_{\text {T}}$ , corresponding to high $\pi ^0$ $p_{\text {T}}$ , the boost of the $\pi ^0$ becomes large enough that the pair of photons almost always creates a single maximum. Figure 4 also shows the probability that a maximum is shared with the other photon in the single $\pi ^0$ sample (share probability).

The $h^{\pm } \,{\ge }2\pi ^0$ decay mode classification is improved by counting the number of maxima associated with $\pi ^0 _\mathrm{cand}$’s. An energy maximum is assigned to a $\pi ^0 _\mathrm{cand}$ if its cell is part of the $\pi ^0 _\mathrm{cand}$ cluster and it has an $E_{\text {T}}$ of more than 300–$430\,\text {MeV}$ (depending on the $\eta$ region). The energy threshold is optimised to maximise the total number of correctly classified tau decays. Maxima with $E_{\text {T}} >10\,\text {GeV}$ are counted twice, as they contain the merged energy deposits of two photons from a $\pi ^0$ decay with a probability larger than 95 %. Finally, $\tau _{\mathrm{had}{\text {-}}\mathrm{vis}}$ candidates that were classified as $h^{\pm } \,\pi ^0$ , but have a $\pi ^0 _\mathrm{cand}$ with at least three associated maxima are reclassified as $h^{\pm } \,{\ge }2\pi ^0$. The method recovers 16 % of misclassified $h^{\pm } \,{\ge }2\pi ^0$ decays with a misclassification of $h^{\pm } \,\pi ^0$ decays of 2.5 %.

### Decay mode classification

Determination of the decay mode by counting the number of reconstructed $h^{\pm }$ ’s and $\pi ^0 _\mathrm{ID}$’s alone can be significantly improved by simultaneously analysing the kinematics of the tau decay products, the $\pi ^0$ identification scores and the number of photons from the previous reconstruction steps. Exploitation of this information is performed via BDTs.

As the most difficult aspect of the classification is to determine the number of $\pi ^0$’s, three decay mode tests are defined to distinguish between the following decay modes: $h^{\pm }$’s with zero or one $\pi ^0$, $h^{\pm } \,\{0,1\}\pi ^0$; $h^{\pm }$’s with one or more $\pi ^0$’s, $h^{\pm } \,\{1,{\ge }2\}\pi ^0$; and 3$h^{\pm }$’s with and without $\pi ^0$’s, $3h^{\pm } \,\{0,{\ge }1\}\pi ^0$. Which of the three tests to apply to a $\tau _{\mathrm{had}{\text {-}}\mathrm{vis}}$ candidate is determined as follows. The $\tau _{\mathrm{had}{\text {-}}\mathrm{vis}}$ candidates with one or three associated tracks without any reconstructed $\pi ^0 _\mathrm{cand}$’s are always classified as $h^{\pm }$ or $3h^{\pm }$ , respectively. The $\tau _{\mathrm{had}{\text {-}}\mathrm{vis}}$ candidates with one associated track and at least two $\pi ^0 _\mathrm{cand}$’s, of which at least one is $\pi ^0 _\mathrm{ID}$, enter the $h^{\pm } \,\{1,{\ge }2\}\pi ^0$ test. The $\tau _{\mathrm{had}{\text {-}}\mathrm{vis}}$ candidates with one $\pi ^0 _\mathrm{ID}$ that are classified as $h^{\pm } \,{\ge }2\pi ^0$ by counting the photons in this cluster, as described in Sect. 3.3, retain their classification and are not considered in the decay mode tests. The remaining $\tau _{\mathrm{had}{\text {-}}\mathrm{vis}}$ candidates with one or three associated tracks enter the $h^{\pm } \,\{0,1\}\pi ^0$ or $3h^{\pm } \,\{0,{\ge }1\}\pi ^0$ tests, respectively.

A BDT is trained for each decay mode test using $\tau _{\mathrm{had}{\text {-}}\mathrm{vis}}$ candidates from simulated $Z \rightarrow \tau \tau$ events, to separate $\tau _{\mathrm{had}{\text {-}}\mathrm{vis}}$’s of the two generated decay types the test is designed to distinguish. The $\tau _{\mathrm{had}{\text {-}}\mathrm{vis}}$ candidates entering each decay mode test are then further categorised based on the number of $\pi ^0 _\mathrm{ID}$’s. A threshold is placed on the output BDT score in each category to determine the decay mode. The thresholds are optimised to maximise the number of correctly classified $\tau _{\mathrm{had}{\text {-}}\mathrm{vis}}$ candidates. The BDT training was not split based on the number of $\pi ^0 _\mathrm{ID}$’s due to the limited size of the training sample.

The variables used for the decay mode tests are designed to discriminate against additional misidentified $\pi ^0 _\mathrm{cand}$’s, which usually come from imperfect $h^{\pm }$ subtraction, pile-up or the underlying event. The associated clusters typically have low energy and a low $\pi ^0$ identification score. Remnant clusters from imperfect $h^{\pm }$ subtraction are also typically close to the $h^{\pm }$ track and have fewer associated photon energy maxima. If the $\pi ^0 _\mathrm{cand}$ clusters originate from tau decays, their directions and fractional energies are correlated with each other. Additionally, with increasing number of tau decay products, the available phase space per decay product becomes smaller. Each variable used in the BDTs is described briefly in Table 4. Table 5 summarises the decay mode tests and indicates which variables are used in each.

**Table 4:** Variables used in the BDTs for the $\tau _{\mathrm{had}{\text {-}}\mathrm{vis}}$ decay mode classification. They are designed to discriminate against additional misidentified $\pi ^0 _\mathrm{cand}$’s, which usually come from imperfect subtraction, pile-up or the underlying event

$\pi ^0$ *identification score of the first* $\pi ^0 _\mathrm{cand}$, $S^\mathrm{BDT}_{1}$
$\pi ^0$ identification score of the $\pi ^0 _\mathrm{cand}$ with the highest $\pi ^0$ identification score
$E_{\text {T}}$ *fraction of the first* $\pi ^0 _\mathrm{cand}$, $f_{\pi ^0,1}$
$E_{\text {T}}$ of the $\pi ^0 _\mathrm{cand}$ with the highest $\pi ^0$ identification score, divided by the $E_{\text {T}}$-sum of all $\pi ^0 _\mathrm{cand}$’s and $h^{\pm }$ ’s
*Hadron separation*, $$\Delta R(h^{\pm },\pi ^0)$$
$\Delta R$ between the $h^{\pm }$ and the $\pi ^0 _\mathrm{cand}$ with the highest $\pi ^0$ identification score
$$h^{\pm }$$ *distance*, $$D_{h^{\pm }}$$
$E_{\text {T}}$-weighted $\Delta R$ between the $h^{\pm }$ and the $\tau _{\mathrm{had}{\text {-}}\mathrm{vis}}$ axis, which is calculated by summing the four-vectors of all $h^{\pm }$ ’s and $\pi ^0 _\mathrm{cand}$’s
*Number of photons*, $N_{\gamma }$
Total number of photons in the $\tau _{\mathrm{had}{\text {-}}\mathrm{vis}}$, as reconstructed in Sect. 3.3
$\pi ^0$ *identification score of second* $\pi ^0 _\mathrm{cand}$, $S^\mathrm{BDT}_{2}$
$\pi ^0$ identification score of the $\pi ^0 _\mathrm{cand}$ with the second-highest $\pi ^0$ identification score
$$\pi ^0 _\mathrm{cand}$$ $$E_{\text {T}}$$ *fraction*, $$f_{\pi ^0}$$
$E_{\text {T}}$-sum of $\pi ^0 _\mathrm{cand}$’s, divided by the $E_{\text {T}}$-sum of $\pi ^0 _\mathrm{cand}$’s and $h^{\pm }$ ’s
$$\pi ^0 _\mathrm{cand}$$ *mass*, $$m_{\pi ^0}$$
Invariant mass calculated from the sum of $\pi ^0 _\mathrm{cand}$ four-vectors
*Number of* $$\pi ^0 _\mathrm{cand}$$, $$N_\mathrm{\pi ^0}$$
*Standard deviation of the* $h^{\pm }$ $p_{\text {T}}$, $\sigma _{E_{\text {T}}, h^{\pm }}$
Standard deviation, calculated from the $p_{\text {T}}$ values of the $h^{\pm }$ ’s for $\tau _{\mathrm{had}{\text {-}}\mathrm{vis}}$ with three associated tracks
$$h^{\pm }$$ *mass*, $$m_{h^{\pm }}$$
Invariant mass calculated from the sum of $h^{\pm }$ four-vectors

**Table 5:** Details regarding the decay mode classification of the Tau Particle Flow. BDTs are trained to distinguish decay modes in three decay mode tests. The $\tau _{\mathrm{had}{\text {-}}\mathrm{vis}}$’s entering each test are further categorised based on the number of reconstructed, $N(\pi ^0 _\mathrm{cand})$, and identified, $N(\pi ^0 _\mathrm{ID})$, neutral pions. The variables used in the BDTs for each test are listed

Decay mode test	$N(\pi ^0 _\mathrm{cand})$	$N(\pi ^0 _\mathrm{ID})$	Variables
$h^{\pm } \,\{0,1\}\pi ^0$	$\ge$1	0	$S^\mathrm{BDT}_{1}$, $f_{\pi ^0,1}$, $\Delta R(h^{\pm },\pi ^0)$, $D_{h^{\pm }}$, $N_{\gamma }$
	1	1	
$h^{\pm } \,\{1,{\ge }2\}\pi ^0$	$\ge$2	1	$S^\mathrm{BDT}_{2}$, $f_{\pi ^0}$, $m_{\pi ^0}$, $N_\mathrm{\pi ^0}$, $N_{\gamma }$
	$\ge 2$	$\ge$2	
$3h^{\pm } \,\{0,{\ge }1\}\pi ^0$	$\ge$1	0	$S^\mathrm{BDT}_{1}$, $f_{\pi ^0}$, $\sigma _{E_{\text {T}}, h^{\pm }}$, $m_{h^{\pm }}$, $N_{\gamma }$
	$\ge 1$	$\ge$1	

**Fig. 5:**
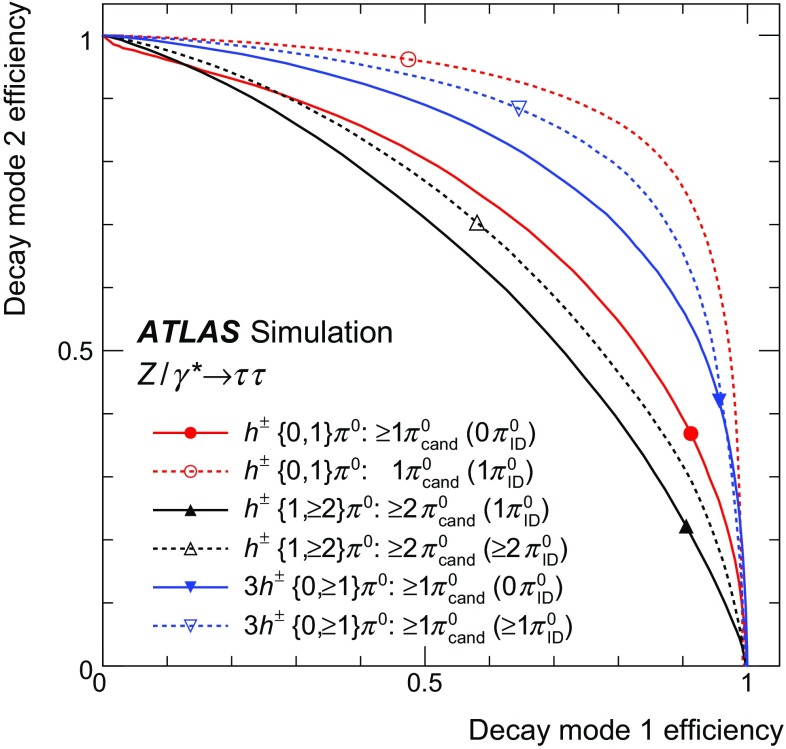
Decay mode classification efficiency for the $h^{\pm } \,\{0,1\}\pi ^0$ , $h^{\pm } \,\{1,{\ge }2\}\pi ^0$ , and $3h^{\pm } \,\{0,{\ge }1\}\pi ^0$ tests. For each test, “decay mode 1” corresponds to the mode with fewer $\pi ^0$’s. Working points corresponding to the optimal thresholds on the BDT score for each test are marked

**Fig. 6:**
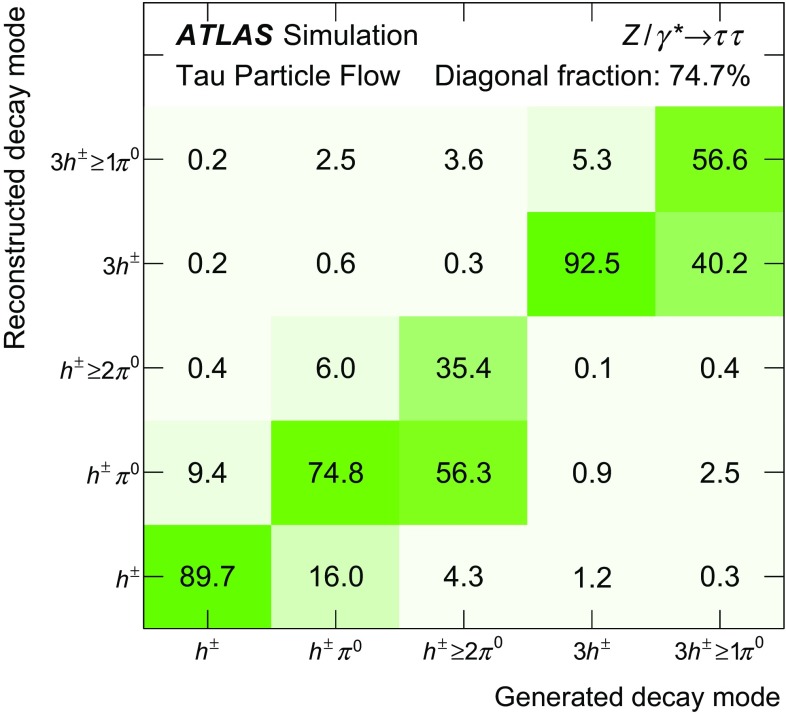
Decay mode classification efficiency matrix showing the probability for a given generated mode to be reconstructed as a particular mode by the Tau Particle Flow after final decay mode classification in simulated $Z \rightarrow \tau \tau$ events. Decays containing neutral kaons are omitted. Only decays from $\tau _{\mathrm{had}{\text {-}}\mathrm{vis}}$’s that are reconstructed and pass the selection described in Sect. 2.2 are considered. The statistical uncertainty is negligible

**Fig. 7:**
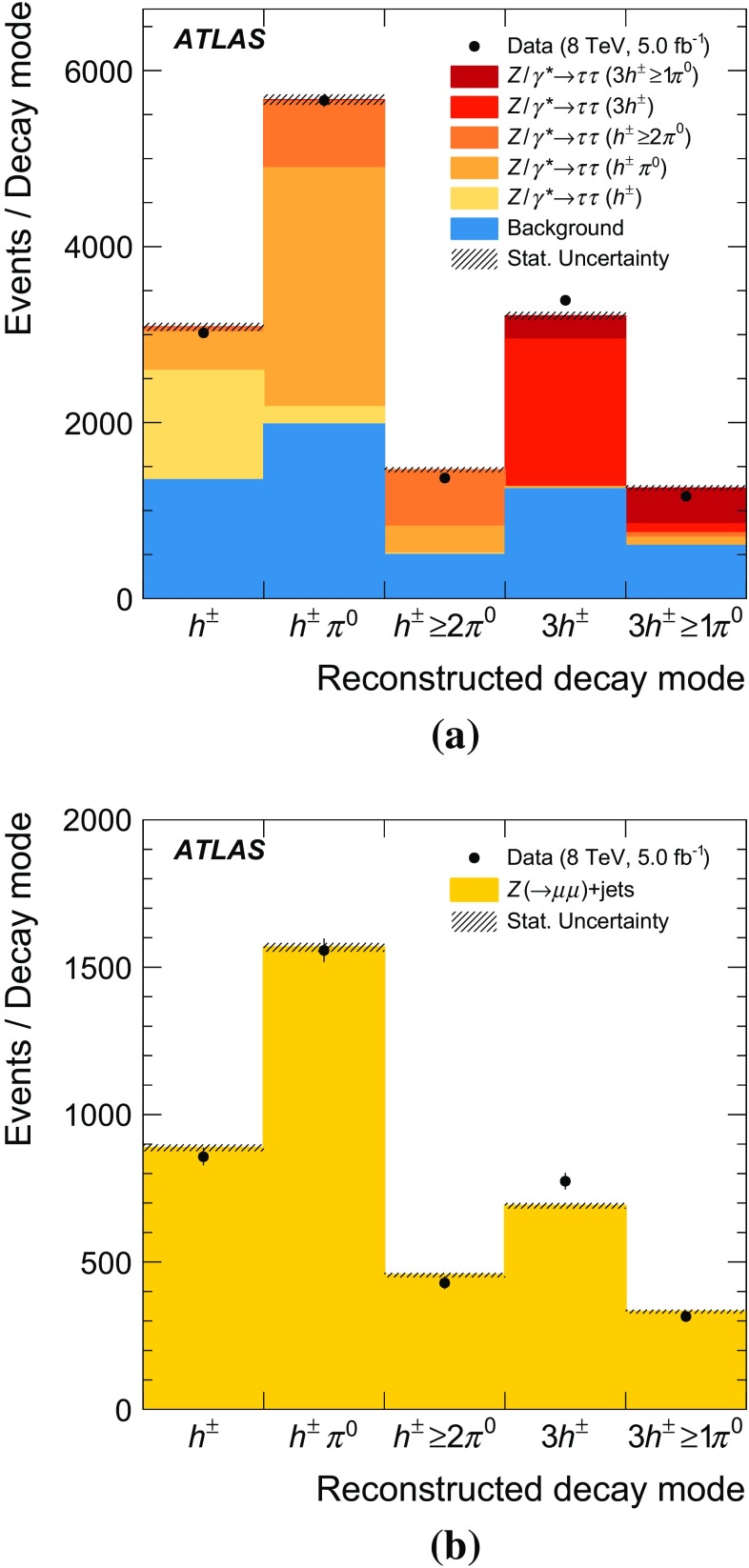
Number of $\tau _{\mathrm{had}{\text {-}}\mathrm{vis}}$ candidates for each classified decay mode in the **a** $Z \rightarrow \tau \tau$ and the **b** $Z(\rightarrow \mu \mu )\mathrm{+jets}$ tag-and-probe analyses. The simulated $Z \rightarrow \tau \tau$ sample is split into contributions from each generated tau decay mode. The background in the $Z \rightarrow \tau \tau$ analysis is dominated by multijet and $W(\rightarrow \mu \nu )\mathrm{+jets}$ production. The simulated $Z(\rightarrow \mu \mu )\mathrm{+jets}$ events are reweighted so that the *Z* boson $p_{\text {T}}$ distribution and the overall normalisation match that in the data. The *hatched band* represents the statistical uncertainty on the prediction

Figure 5 shows the discrimination power of the tests categorised by the number of $\pi ^0 _\mathrm{cand}$’s and $\pi ^0 _\mathrm{ID}$’s. The decay mode fractions at the input of each test vary strongly, which impacts the position of the optimal BDT requirements. The resulting classification matrix is shown in Fig. 6. The total fraction of correctly classified tau decays is 74.7 %. High efficiencies in the important $h^{\pm }$, $h^{\pm } \,\pi ^0$ and $3h^{\pm }$ modes are achieved. The *decay mode purity* is defined as the fraction of $\tau _{\mathrm{had}{\text {-}}\mathrm{vis}}$ candidates of a given reconstructed mode which originated from a generated $\tau _{\mathrm{had}{\text {-}}\mathrm{vis}}$ of the same mode, also calculated using $\tau _{\mathrm{had}{\text {-}}\mathrm{vis}}$’s in simulated $Z \rightarrow \tau \tau$ events. The purity of the $h^{\pm }$, $h^{\pm } \,\pi ^0$ and $3h^{\pm }$ decay modes is 70.3, 73.5 and 85.2 %, respectively. For comparison, in the Baseline reconstruction where $\pi ^0$ reconstruction was not available, the fractions of generated $h^{\pm }$ and $h^{\pm } \,\pi ^0$ in $\tau _{\mathrm{had}{\text {-}}\mathrm{vis}}$’s with one reconstructed track are 27.4 and 52.2 %, respectively, and the fraction of $3h^{\pm }$ in $\tau _{\mathrm{had}{\text {-}}\mathrm{vis}}$’s with three reconstructed tracks is 68.9 %. Decays containing neutral kaons are omitted from the table. They are classified as containing $\pi ^0$’s approximately half of the time. Contributions from pile-up have little effect on the classification efficiency, degrading it by $\sim$0.04 % per additional reconstructed vertex. The number of $\tau _{\mathrm{had}{\text {-}}\mathrm{vis}}$ candidates for each classified decay mode is shown in Fig. 7a for real $\tau _{\mathrm{had}{\text {-}}\mathrm{vis}}$’s from the $Z \rightarrow \tau \tau$ tag-and-probe analysis and in Fig. 7b for jets from the $Z(\rightarrow \mu \mu )\mathrm{+jets}$ tag-and-probe analysis. While systematic uncertainties have not been evaluated, the figures indicate reasonable modelling of the decay mode classification for $\tau _{\mathrm{had}{\text {-}}\mathrm{vis}}$’s and jets. In both selections, the $3h^{\pm }$ efficiency is slightly underestimated and the $h^{\pm } \,{\ge }2\pi ^0$ and $3h^{\pm } \,{\ge }1\pi ^0$ efficiencies are slightly overestimated.

### Four-momentum reconstruction

**Fig. 8:**
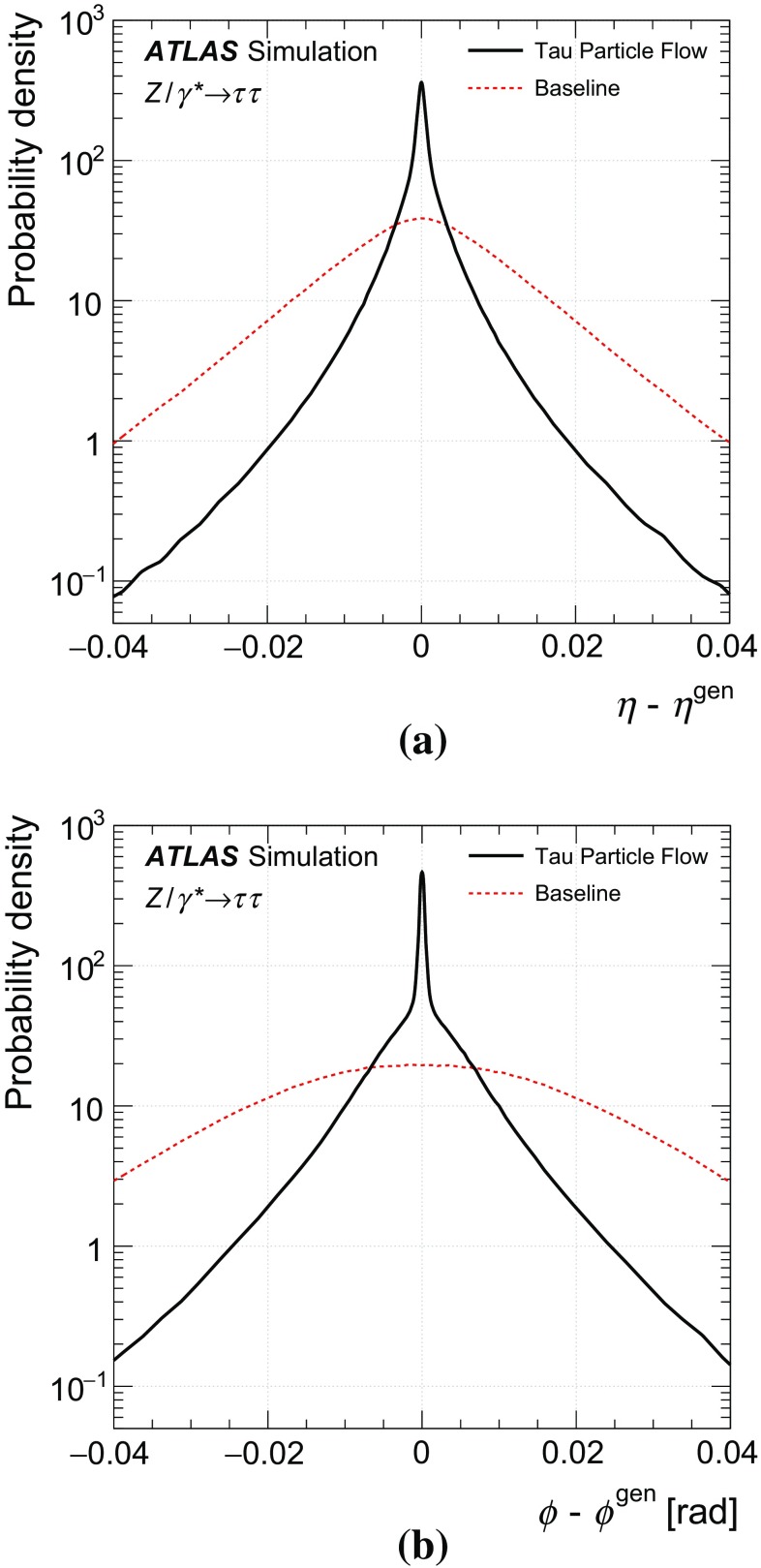
The $\tau _{\mathrm{had}{\text {-}}\mathrm{vis}}$ **a** $\eta$ and **b** $\phi$ residual distributions of the Tau Particle Flow compared to the Baseline reconstruction

The $\tau _{\mathrm{had}{\text {-}}\mathrm{vis}}$ four-momentum reconstruction begins with summing the four-momenta of the $h^{\pm }$ and $\pi ^0 _\mathrm{cand}$ constituents (Constituent-based calculation). Only the first *n* $\pi ^0 _\mathrm{cand}$’s with the highest $\pi ^0$ identification scores are included, where *n* is determined from the decay mode classification, and can be at most 2 $\pi ^0 _\mathrm{cand}$’s in the $h^{\pm } \,{\ge }2\pi ^0$ mode and at most 1 $\pi ^0 _\mathrm{cand}$ in the $3h^{\pm } \,{\ge }1\pi ^0$ mode. A pion mass hypothesis is used for $\pi ^0 _\mathrm{cand}$’s. There are two exceptions: if the decay mode is classified as $h^{\pm } \,\pi ^0$ but there are two identified $\pi ^0 _\mathrm{cand}$’s, the mass of each is set to zero and both are added to the $\tau _{\mathrm{had}{\text {-}}\mathrm{vis}}$ four-momentum as they are most likely photons from a $\pi ^0$ decay; or if the $\tau _{\mathrm{had}{\text {-}}\mathrm{vis}}$ candidate is classified as $h^{\pm } \,{\ge }2\pi ^0$ because three or more photons are found in a single $\pi ^0 _\mathrm{cand}$, only this $\pi ^0 _\mathrm{cand}$ is added and its mass is set to twice the $\pi ^0$ mass. A calibration is applied to the Constituent-based $\tau _{\mathrm{had}{\text {-}}\mathrm{vis}}$ energy in each decay mode as a function of the Constituent-based $E_{\text {T}}$ , to correct for the $\pi ^0 _\mathrm{cand}$ energy bias. The resulting four-momentum is used to set the $\tau _{\mathrm{had}{\text {-}}\mathrm{vis}}$ direction in the Tau Particle Flow. Figure 8a, b show distributions of the $\tau _{\mathrm{had}{\text {-}}\mathrm{vis}}$ $\eta$ and $\phi$ residuals of the Tau Particle Flow and the Baseline four-momentum reconstruction. The core angular resolutions of the Tau Particle Flow are 0.002 in $\eta$ and 0.004 rad in $\phi$, which are more than five times better than the Baseline resolutions of 0.012 and 0.02 rad, respectively.

**Fig. 9:**
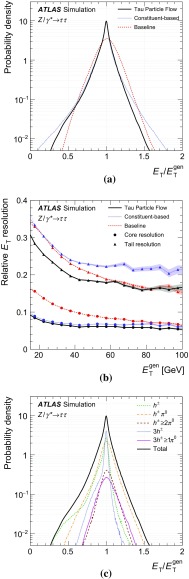
The **a** $\tau _{\mathrm{had}{\text {-}}\mathrm{vis}}$ relative $E_{\text {T}}$ residual distribution and **b** the half-widths spanned by the 68 and 95 % quantiles, i.e. the core and tail resolutions, of the relative $E_{\text {T}}$ residual distributions as a function of the generated $\tau _{\mathrm{had}{\text {-}}\mathrm{vis}}$ $E_{\text {T}}$. The Baseline, Constituent-based and Tau Particle Flow calculations are shown. The relative $E_{\text {T}}$ residual distribution of the Tau Particle Flow split in the reconstructed decay mode **c** is also shown

Figure 9a shows distributions of the $E_{\text {T}}$ residuals. The Constituent-based calculation is inherently stable against pile-up as both the decay-mode classification used to select $h^{\pm }$ ’s and $\pi ^0 _\mathrm{cand}$’s, and the reconstruction of $h^{\pm }$ ’s and $\pi ^0 _\mathrm{cand}$’s themselves, are stable against pile-up. The $E_{\text {T}}$ increases by $\sim$6$\,\text {MeV}$ and its resolution degrades fractionally by $\sim$0.6 % per additional reconstructed vertex. Figure 9b shows the resolution as a function of the $E_{\text {T}}$ of the generated $\tau _{\mathrm{had}{\text {-}}\mathrm{vis}}$ . For the final energy calibration of the Tau Particle Flow, the Constituent-based $E_{\text {T}}$ is combined with the Baseline $E_{\text {T}}$ by weighting each by the inverse-square of their respective $E_{\text {T}}$-dependent core resolutions, which ensures a smooth transition to high $p_{\text {T}}$ where the Baseline calibration is superior. The Baseline $E_{\text {T}}$ is used if the two $E_{\text {T}}$ values disagree by more than five times their combined core resolutions, as it has smaller resolution tails. The resolution of the Tau Particle Flow is superior in both the core and tails at low $E_{\text {T}}$ with a core resolution of 8 % at an $E_{\text {T}}$ of 20 $\,\text {GeV}$, compared to 15 % from the Baseline. It approaches the Baseline performance at high $E_{\text {T}}$ . Contributions from pile-up have little effect on the four-momentum reconstruction of the Tau Particle Flow; the $E_{\text {T}}$ increases by $\sim$4$\,\text {MeV}$ and its core resolution degrades fractionally by $\sim$0.5 % per additional reconstructed vertex. The $E_{\text {T}}$ residual distributions of the Tau Particle Flow split into the reconstructed decay modes are shown in Fig. 9c. The total is non-Gaussian, as it is the sum of contributions with different functional forms. Correctly reconstructed decays containing only $h^{\pm }$ ’s have the best resolution, followed by correctly reconstructed decays containing $\pi ^0 _\mathrm{cand}$’s. The excellent resolution of these decays leads to a superior overall core resolution. Misreconstructed decays have the poorest resolution and result in larger tails. In particular, misestimation of the number of $\pi ^0 _\mathrm{cand}$’s leads to a bias of up to 25 %. Decays containing neutral kaons exhibit a large low-energy bias because at least some of their energy is typically missed by the reconstruction.

An alternative method for the $E_{\text {T}}$ calibration was also developed, based on Ref. [30]. It also uses a combination of calorimetric and tracking measurements and the Tau Particle Flow decay mode classification. The $h^{\pm }$ $p_{\text {T}}$ is measured using tracks and the $\pi ^0$ $E_{\text {T}}$ is estimated as the difference between the $E_{\text {T}}$ of the seed jet at the EM scale [36] and the $E_{\text {T}}$ from the summed momenta of all $h^{\pm }$ ’s, scaled by their expected calorimeter response [60]. The method has similar overall performance to the Tau Particle Flow.

**Fig. 10:**
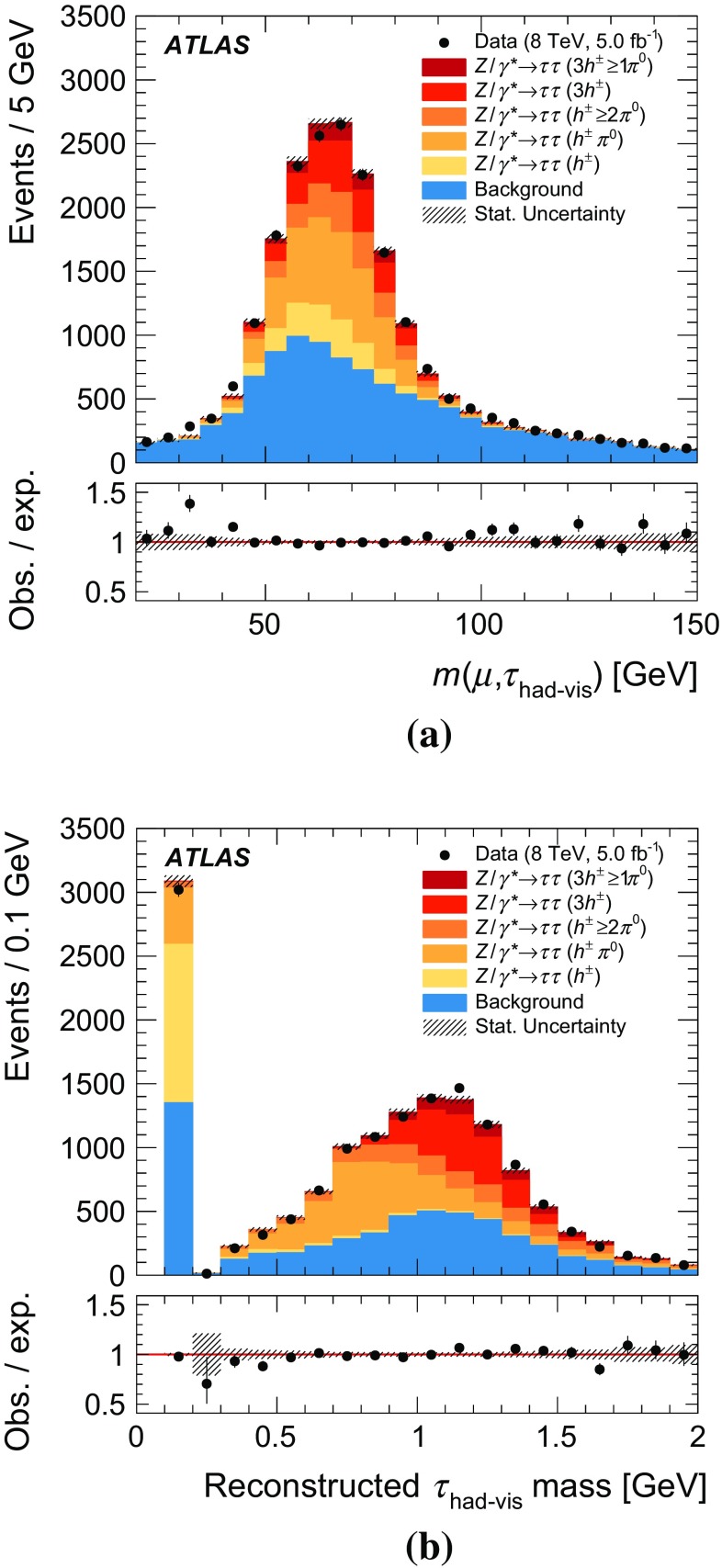
Distribution of **a** the invariant mass of the muon and $\tau _{\mathrm{had}{\text {-}}\mathrm{vis}}$, $m(\mu ,\tau _{\mathrm{had}{\text {-}}\mathrm{vis}})$ before selection on $m(\mu ,\tau _{\mathrm{had}{\text {-}}\mathrm{vis}})$ is applied; and **b** the reconstructed mass of the $\tau _{\mathrm{had}{\text {-}}\mathrm{vis}}$ , when using the Tau Particle Flow $\tau _{\mathrm{had}{\text {-}}\mathrm{vis}}$ four-momentum reconstruction in the $Z \rightarrow \tau \tau$ tag-and-probe analysis. The simulated $Z \rightarrow \tau \tau$ sample is split into contributions from each generated tau decay mode. The background is dominated by multijet and $W(\rightarrow \mu \nu )\mathrm{+jets}$ production. The hatched band represents the statistical uncertainty on the prediction

Figure 10a shows the distribution of the invariant mass of the muon and $\tau _{\mathrm{had}{\text {-}}\mathrm{vis}}$, $m(\mu ,\tau _{\mathrm{had}{\text {-}}\mathrm{vis}})$, calculated using the $\tau _{\mathrm{had}{\text {-}}\mathrm{vis}}$ four-momentum reconstruction from the Tau Particle Flow in the $Z \rightarrow \tau \tau$ tag-and-probe analysis before selection on $m(\mu ,\tau _{\mathrm{had}{\text {-}}\mathrm{vis}})$. The $m(\mu ,\tau _{\mathrm{had}{\text {-}}\mathrm{vis}})$ has a linear dependence on the $\tau _{\mathrm{had}{\text {-}}\mathrm{vis}}$ $E_{\text {T}}$ and analysis of the distribution has previously been used to calibrate the $\tau _{\mathrm{had}{\text {-}}\mathrm{vis}}$ $E_{\text {T}}$  [17]. Data and simulation agree well, indicating that the $\tau _{\mathrm{had}{\text {-}}\mathrm{vis}}$ $E_{\text {T}}$ is well modelled by the simulation. Finally, Fig. 10b shows the mass spectrum of the $\tau _{\mathrm{had}{\text {-}}\mathrm{vis}}$ reconstructed with the Tau Particle Flow in the $Z \rightarrow \tau \tau$ tag-and-probe analysis. The $a_1$ resonance in the $3h^{\pm }$ mode is reconstructed with negligible experimental resolution compared to the intrinsic line shape due to the excellent four-momentum resolution of the inner detector for $h^{\pm }$ ’s. The $\rho$ and $a_1$ resonances in the $h^{\pm } \,\pi ^0$ and $h^{\pm } \,{\ge }2\pi ^0$ modes are also visible, but have significant degradation due to the resolution from the reconstructed $\pi ^0 _\mathrm{cand}$ four-momentum. The $\tau _{\mathrm{had}{\text {-}}\mathrm{vis}}$ mass spectra in data and simulation agree well, suggesting good modelling of the individual $h^{\pm }$ and $\pi ^0 _\mathrm{cand}$ four-momenta.

## Summary and conclusions

This paper presents a new method to reconstruct the individual charged and neutral hadrons in tau decays with the ATLAS detector at the LHC. The neutral pions are reconstructed with a core energy resolution of $\sim$16 %. The reconstructed hadrons are used to calculate the visible four-momentum of reconstructed tau candidates and to classify the decay mode, allowing the decays to be distinguished not only by the number of $h^{\pm }$ ’s but also by the number of $\pi ^0$’s, which is not possible with the existing tau reconstruction. This improves the purity with which the $\tau ^{-} \rightarrow \pi ^{-} \nu$, $\tau ^{-} \rightarrow \pi ^{-} \pi ^0 \nu$ and $\tau ^{-} \rightarrow \pi ^{-} \pi ^+ \pi ^{-} \nu$ decays can be selected, by factors of 2.6, 1.4 and 1.2, respectively. The $\tau _{\mathrm{had}{\text {-}}\mathrm{vis}}$ core directional resolution is improved by more than a factor of five and the core energy resolution is improved by up to a factor of two at low $E_{\text {T}}$ ($20\,\text {GeV}$). The performance was validated using samples of $Z \rightarrow \tau \tau$ and $Z(\rightarrow \mu \mu )\mathrm{+jets}$ events selected from *pp* collision data at $\sqrt{s}=8\,\text {TeV}$, corresponding to an integrated luminosity of 5 $\mathrm{fb}^{-1}$. The results suggest good modelling of the $\tau _{\mathrm{had}{\text {-}}\mathrm{vis}}$ decay mode classification efficiency and four-momentum reconstruction.

## Footnotes

1 ATLAS uses a right-handed coordinate system with its origin at the nominal interaction point (IP) in the centre of the detector and the *z*-axis along the beam direction. The *x*-axis points from the IP to the centre of the LHC ring, and the *y*-axis points upward. Cylindrical coordinates $(r,\phi )$ are used in the transverse (*x*, *y*) plane, $\phi$ being the azimuthal angle around the beam direction. The pseudorapidity is defined in terms of the polar angle $\theta$ as $\eta =-\ln \tan (\theta /2)$. The distance $\Delta {}R$ in the $\eta$–$\phi$ space is defined as $\Delta {}R=\sqrt{({\Delta \eta })^2 + ({\Delta \phi })^2}$.

## References

[1] ATLAS Collaboration, The ATLAS experiment at the CERN large hadron collider. JINST **3**, S08003 (2008)

[2] Evans L, Bryant P, Machine LHC (2008). JINST.

[3] ATLAS Collaboration, Measurement of the $$t\bar{t}$$ production cross section in the tau+jets channel using the ATLAS detector. Eur. Phys. J. C **73**, 2328 (2013). arXiv:1211.7205 [hep-ex] 10.1140/epjc/s10052-013-2328-7 PMC4371093 25814855

[4] ATLAS Collaboration, Measurement of the top quark pair cross section with ATLAS in $$pp$$ collisions at $$\sqrt{s} = 7\;\text{ TeV }$$ using final states with an electron or a muon and a hadronically decaying $$\tau $$ lepton. Phys. Lett. B **717**, 89 (2012). arXiv:1205.2067 [hep-ex]

[5] ATLAS Collaboration, Measurement of the $$W \rightarrow \tau \nu _{\tau }$$ cross section in $$pp$$ collisions at $$\sqrt{s} = 7\;\text{ TeV }$$ with the ATLAS experiment. Phys. Lett. B **706**, 276 (2012). arXiv:1108.4101 [hep-ex]

[6] ATLAS Collaboration, Measurement of $$\tau $$ polarization in $$W \rightarrow \tau \nu $$ decays with the ATLAS detector in $$pp$$ collisions at $$\sqrt{s} = 7\;\text{ TeV }$$. Eur. Phys. J. C **72**, 2062 (2012). arXiv:1204.6720 [hep-ex] 10.1140/epjc/s10052-012-2062-6 PMC4371045 25814842

[7] ATLAS Collaboration, Measurement of the $$Z \rightarrow \tau \tau $$ cross section with the ATLAS detector. Phys. Rev. D **84**, 112006 (2011). arXiv:1108.2016 [hep-ex]

[8] ATLAS Collaboration, Evidence for the Higgs-boson Yukawa coupling to tau leptons with the ATLAS detector. JHEP **1504**, 117 (2015). arXiv:1501.04943 [hep-ex]

[9] ATLAS Collaboration, Search for charged Higgs bosons through the violation of lepton universality in $$t\bar{t}$$ events using $$pp$$ collision data at $$\sqrt{s} = 7\;\text{ TeV }$$ with the ATLAS experiment. JHEP **1303**, 076 (2013). arXiv:1212.3572 [hep-ex]

[10] ATLAS Collaboration,Search for charged Higgs bosons decaying via $$H^{\pm } \rightarrow \tau \nu $$ in $$t\bar{t}$$ events using $$pp$$ collision data at $$\sqrt{s} = 7\;\text{ TeV }$$ with the ATLAS detector. JHEP **1206**, 039 (2012). arXiv:1204.2760 [hep-ex]

[11] ATLAS Collaboration, Search for the neutral Higgs bosons of the minimal supersymmetric standard model in $$pp$$ collisions at $$\sqrt{s} = 7\;\text{ TeV }$$ with the ATLAS detector. JHEP **1302**, 095 (2013). arXiv:1211.6956 [hep-ex]

[12] ATLAS Collaboration, Search for supersymmetry in events with large missing transverse momentum, jets, and at least one tau lepton in $$20\;\text{ fb }^{-1}$$ of $$\sqrt{s} = 8\;\text{ TeV }$$ proton–proton collision data with the ATLAS detector. JHEP **1409**, 103 (2014). arXiv:1407.0603 [hep-ex]

[13] ATLAS Collaboration, Search for the direct production of charginos, neutralinos and staus in final states with at least two hadronically decaying taus and missing transverse momentum in $$pp$$ collisions at $$\sqrt{s} = 8\;\text{ TeV }$$ with the ATLAS detector. JHEP **1410**, 096 (2014). arXiv:1407.0350 [hep-ex]

[14] ATLAS Collaboration, Search for a heavy narrow resonance decaying to $$e \mu $$, $$e \tau $$, or $$\mu \tau $$ with the ATLAS detector in $$\sqrt{s} = 7\;\text{ TeV }$$$$pp$$ collisions at the LHC. Phys. Lett. B **723**, 15 (2013). arXiv:1212.1272 [hep-ex]

[15] ATLAS Collaboration, A search for high-mass resonances decaying to $$\tau ^+\tau ^-$$ in $$pp$$ collisions at $$\sqrt{s} = 7\;\text{ TeV }$$ with the ATLAS detector. Phys. Lett. B **719**, 242 (2013). arXiv:1210.6604 [hep-ex]

[16] ATLAS Collaboration, Search for third generation scalar leptoquarks in $$pp$$ collisions at $$\sqrt{s} = 7\;\text{ TeV }$$ with the ATLAS detector. JHEP **1306**, 033 (2013). arXiv:1303.0526 [hep-ex]

[17] ATLAS Collaboration, Identification and energy calibration of hadronically decaying tau leptons with the ATLAS experiment in $$pp$$ collisions at $$\sqrt{s}=8{\text{ TeV }}$$. Eur. Phys. J. C **75**, 303 (2015). arXiv:1412.7086 [hep-ex] 10.1140/epjc/s10052-015-3500-z PMC4498687 26190938

[18] ATLAS Collaboration, Observation of a new particle in the search for the Standard Model Higgs boson with the ATLAS detector at the LHC. Phys. Lett. B **716**, 1 (2012). arXiv:1207.7214 [hep-ex]

[19] CMS Collaboration, Observation of a new boson at a mass of 125 GeV with the CMS experiment at the LHC. Phys. Lett. B **716**, 30 (2012). arXiv:1207.7235 [hep-ex]

[20] CMS Collaboration, Evidence for the 125 GeV Higgs boson decaying to a pair of $$\tau $$ leptons. JHEP **1405**, 104 (2014). arXiv:1401.5041 [hep-ex]

[21] Desch K (2004). Probing the CP nature of the Higgs boson at linear colliders with tau spin correlations: the case of mixed scalar—pseudoscalar couplings. Phys. Lett. B.

[22] R. Harnik et al., Measuring CP violation in $$h \rightarrow \tau ^+ \tau ^-$$ at colliders. Phys. Rev. D **88**, 076009 (2013). arXiv:1308.1094 [hep-ph]

[23] Berge S, Bernreuther W, Kirchner S (2015). Prospects of constraining the Higgs boson’s CP nature in the tau decay channel at the LHC. Phys. Rev. D.

[24] Elagin A (2011). A new mass reconstruction technique for resonances decaying to di-tau. Nucl. Instrum. Meth. A.

[25] ALEPH Collaboration, S. Schael et al., Branching ratios and spectral functions of tau decays: final ALEPH measurements and physics implications. Phys. Rept. **421**, 191 (2005). arXiv:hep-ex/0506072 [hep-ex]

[26] OPAL Collaboration, R. Akers et al., Measurement of the $$\tau ^{-}\rightarrow {h}^{-}{\pi ^{0}}\nu _{\tau }$$ and $$\tau ^{-} \rightarrow {h}^{-} \ge 2{\pi ^{0}} \nu _{\tau }$$ branching ratios. Phys. Lett. B **328**, 207 (1994)

[27] DELPHI Collaboration, J. Abdallah et al., A Measurement of the tau hadronic branching ratios. Eur. Phys. J. C **46**, 1 (2006). arXiv:hep-ex/0603044 [hep-ex]

[28] L3 Collaboration, M. Acciarri et al., Measurement of tau polarization at LEP. Phys. Lett. B **429**, 387 (1998)

[29] Elagin A (2013). Probabilistic particle flow algorithm for high occupancy environment. Nucl. Instrum. Meth. A.

[30] D0 Collaboration, V.M. Abazov et al., Measurement of $$\sigma (p\bar{p} \rightarrow Z + X)$$ Br($$Z \rightarrow \tau ^+ \tau ^-$$) at $$\sqrt{s}$$ = 1.96 TeV. Phys. Lett. B **670**, 292 (2009). arXiv:0808.1306 [hep-ex]

[31] CMS Collaboration, Reconstruction and identification of $$\tau $$ lepton decays to hadrons and $$\nu _{\tau }$$ at CMS. JINST **11**, P01019 (2016). arXiv:1510.07488 [physics.ins-det]

[32] ATLAS Collaboration, Performance of the ATLAS trigger system in 2010. Eur. Phys. J. C **72**, 1849 (2012). arXiv:1110.1530 [hep-ex]

[33] Cacciari M, Salam GP, Soyez G (2008). The anti-$$k_{t}$$ jet clustering algorithm. JHEP.

[34] Cacciari M, Salam GP (2006). Dispelling the $$N^{3}$$ myth for the $$k_{t}$$ jet-finder. Phys. Lett. B.

[35] W. Lampl et al., Calorimeter Clustering Algorithms: Description and Performance, ATL-LARG-PUB-2008-002 (2008). http://cds.cern.ch/record/1099735

[36] T. Barillari et al., Local hadronic calibration, ATL-LARG-PUB-2009-001 (2009). http://cds.cern.ch/record/1112035

[37] A. Hoecker et al., TMVA: toolkit for multivariate data analysis. PoS **ACAT**, 040 (2007). arXiv:physics/0703039

[38] ATLAS Collaboration, Measurement of the muon reconstruction performance of the ATLAS detector using 2011 and 2012 LHC protona–proton collision data. Eur. Phys. J. C **74**, 3130 (2014). arXiv:1407.3935 [hep-ex] 10.1140/epjc/s10052-014-3130-x PMC4371046 25814875

[39] ATLAS Collaboration, Performance of missing transverse momentum reconstruction in proton–proton collisions at $$\sqrt{s} = 7\;\text{ TeV }$$ with ATLAS. Eur. Phys. J. C **72**, 1844 (2012). arXiv:1108.5602 [hep-ex]

[40] Ilten P (2014). Tau decays in pythia 8. Nucl. Phys. B.

[41] Davidson N (2012). Universal interface of TAUOLA: technical and physics documentation. Comput. Phys. Commun..

[42] Golonka P, Was Z (2006). PHOTOS Monte Carlo: a precision tool for QED corrections in Z and W decays. Eur. Phys. J. C.

[43] Sjöstrand T, Mrenna S, Skands P (2008). A brief introduction to PYTHIA 8.1. Comput. Phys. Commun..

[44] Nadolsky PM (2008). Implications of CTEQ global analysis for collider observables. Phys. Rev. D.

[45] ATLAS Collaboration, Summary of ATLAS Pythia 8 tunes. ATL-PHYS-PUB-2012-003 (2012). http://cds.cern.ch/record/1474107

[46] Mangano ML (2003). ALPGEN, a generator for hard multiparton processes in hadronic collisions. JHEP.

[47] Skands PZ (2010). Tuning Monte Carlo generators: the perugia tunes. Phys. Rev. D.

[48] Frixione S, Webber BR (2002). Matching NLO QCD computations and parton shower simulations. JHEP.

[49] Frixione S (2006). Single-top production in MCNLO. JHEP.

[50] Frixione S (2008). Single-top hadroproduction in association with a W boson. JHEP.

[51] Corcella G (2001). HERWIG 6: an event generator for hadron emission reactions with interfering gluons (including supersymmetric processes). JHEP.

[52] Butterworth JM, Forshaw JR, Seymour MH (1996). Multiparton interactions in photoproduction at HERA. Z. Phys. C..

[53] Lai H-L (2010). New parton distributions for collider physics. Phys. Rev. D.

[54] ATLAS Collaboration, The ATLAS simulation infrastructure. Eur. Phys. J. C **70**, 823 (2010). arXiv:1005.4568 [hep-ex]

[55] GEANT4 Collaboration, S. Agostinelli et al., Geant4—a simulation toolkit. Nucl. Instr. Meth. A **506**, 250–303 (2003)

[56] G. Folger, J.P. Wellisch, String parton models in Geant4 (2003). arXiv:nucl-th/0306007

[57] Bertini HW (1969). Intranuclear-cascade calculation of the secondary nucleon spectra from nucleon-nucleus interactions in the energy range 340 to 2900 MeV and comparisons with experiment. Phys. Rev..

[58] ATLAS Collaboration, Measurement of the production cross section of jets in association with a $$Z$$ boson in $$pp$$ collisions at $$\sqrt{s} = 7\;\text{ TeV }$$ with the ATLAS detector. JHEP **1307**, 032 (2013). arXiv:1304.7098 [hep-ex]

[59] Olive K (2014). Review of particle physics. Chin. Phys. C.

[60] ATLAS Collaboration, A measurement of single hadron response using data at $$\sqrt{s} = 8\;\text{ TeV }$$ with the ATLAS detector. ATL-PHYS-PUB-2014-002 (2014). http://cdsweb.cern.ch/record/1668961

